# Fractional shot noise of an SU(*N*) Kondo system

**DOI:** 10.3762/bjnano.17.34

**Published:** 2026-04-14

**Authors:** Damian Krychowski, Stanisław Lipiński

**Affiliations:** 1 Department of Theory of Nanostructures and Quantum Materials, Institute of Molecular Physics, Polish Academy of Sciences, M. Smoluchowskiego 17, 60-179 Poznań, Poland

**Keywords:** Fermi liquid, Kondo effect, quantum dots, shot noise

## Abstract

We consider transport through a multilevel interacting quantum dot (N-QD) in the Kondo regime. Using the Kotliar–Ruckentein slave boson approach (SBMFA) for an *N*-level Anderson model, we define effectively noninteracting quasiparticles of the SU(*N*) Kondo system (*N* = 2, 3, 4, 5, 6). Kondo resonance transmission coefficients determine linear noise describing quasiparticle partitioning. To discuss nonlinear conductance, susceptibilities, and shot noise in the strong coupling regime, we apply Fermi liquid theory with parameters expressed by susceptibilities of pseudofermions determined within SBMFA. Nonlinear shot noise is dominated by two-quasiparticle scattering. However, we demonstrate that for occupation regions distant from the electron–hole symmetry point, the role of three-body correlations must be considered.

## Introduction

Quantum dot (QD) structures are being intensively investigated regarding both fundamental physics and potential quantum information applications, detection, and sensing [1–4]. To achieve these goals, the increasing ability to manipulate quantum states is crucial. As electrons are confined in fewer dimensions and as the size of the dot decreases, the charging energy of a single excess charge on the dot increases. Strong dynamic correlations start to play a dominant role when Coulomb interaction exceeds electron kinetic energy. For dots weakly coupled to the leads, many-body resonances build up at low temperatures, opening new paths for coherent transport. Due to the tunability of QDs by external fields, voltages, and strains, strong correlations can be tested also in regimes not accessible in solid-state physics. The paramagnetic SU(2) Kondo effect has been observed in semiconductor-based QDs [5–6], in carbon nanotubes (CNTs) [7–11], and in molecular structures [12–13]. In this phenomenon, an entangled state is formed as a result of screening of the localized spin by conduction electrons [14–16]. The growing interest in the Kondo effect in nanoscopic systems is motivated primarily by cognitive purposes as this phenomenon is a basis for understanding a large variety of intricate many-body problems. Potential applications are also relevant. Let us just mention a few: The Kondo effect can be used, for example, as conductance control mechanism [5,17], in probing magnetic interactions [18], or, when polarized electrodes are connected, also for the generation of spin-polarized currents [19–23]. A continuing present goal is to test fundamental correlations between different degrees of freedom and to examine their role in quantum transport. The principal aim is to understand how dot or multidot structures with internal spin, orbital, or charge isospin may lead to variants of the high-symmetry Kondo effect involving different degrees of freedom [24–32]. Spin and orbital degeneracies can occur simultaneously, leading to a Kondo ground state of SU(4) symmetry with orbital–spin entanglement. The simultaneous screening of charge or orbital pseudospin and the real spin has been reported in vertical QDs [30], in capacitively coupled dots [24], and in CNTs [29]. Suggestions for the realization of SU(3) Kondo physics can be found, for example, in [33–35]. One of the interesting directions of the study of the Kondo effect is to incorporate higher-rank SU(*N*) symmetries (*N >* 2). The first SU(*N*) generalizations of the Anderson model appeared in the literature on heavy fermion systems [36], where large *N* expansion proved to be a powerful approximation for describing magnetic atoms with orbital degeneracy. From the perspective of potential applications, it is important that the Anderson SU(*N*) model can be realized in a controlled way in various nanoscopic structures [37–38] and in correlated cold atomic gases [39–40]. A proposal of the SU(6) Kondo effect for a QD structure can be found in [39] and for cold atoms in [40]. There are also reports on Kondo effects for *N >* 6, for example, the SU(12) Kondo effect in carbon nanotubes has been analyzed in [41]. In the SU(*N*) case, the system has *N* flavors instead of spin-up and spin-down, and the three Pauli matrices are generalized to (*N*^2^ − 1) generators for an *N*-dimensional space of the Lie group [42–44]. Recently, interest in quantum simulators, engineered quantum many-body systems that can controllably simulate complex quantum phenomena, has rapidly risen. Multilevel dots or dot systems provide a versatile platform for such simulations addressing questions across various domains of condensed matter and field theory [45–48]. Using spin or charge Kondo building blocks could eventually lead to quantum simulations of exotic lattice models, including those almost unrealistic in solid-state systems. The motivation for studies of high-symmetry Kondo effects in nanostructures is the fact that Kondo temperatures dramatically increase with degeneracy and take on values that are experimentally accessible [15]. At the same time, the Kondo resonance peak remains narrower than Coulomb resonances. This makes SU(*N*) Kondo systems attractive for transport control because, in the Kondo regime, transport characteristics remain narrow with respect to the gate voltage.

In this work, we limit ourselves to a discussion of thermodynamic and transport properties of the basic unitary symmetries SU(*N*) *N* = 2, 3, 4, 5, 6 that one encounters in nanoscopic systems. We address our calculations to multilevel two-dimensional electron gas (2DEG) quantum dots [49] and to single-walled and multiwalled carbon nanotube QDs [50–51]. It is worth mentioning that these symmetries are of a fundamental nature and concern systems from various, sometimes very distant, fields of physics, including chromodynamics or Grand Unified Theories [52]. Conductance, the most fundamental transport property, provides information on the time-averaged electron transport. In a rough picture, this quantity can be understood by a Landauer–Büttiker-type form, where conductance is expressed by transmission, for the case of interacting electrons appropriately renormalized [53]. For Kondo-correlated systems, it is the many-body Kondo resonance forming at the Fermi level that enables perfect electron transmission. In the Kondo regime, conductance and many other quantities exhibit universal scaling, the Kondo temperature *k*_B_*T*_K_ being the only energy scale that governs low-energy properties [54]. The Kondo temperature can be extracted, for example, from the temperature dependence of conductance or from the susceptibility [4,55–56]. For bulk systems, also spectroscopic measurements of temperature evolution of Kondo peaks provide this information [57]. Although some information about correlations is hidden in the renormalization of transmission, a deeper insight into the correlations of electronic wave functions can be obtained from the shot noise; therefore, we especially focus in this paper on the analysis of this quantity. Despite high theoretical interest [58–59], relatively little experimental studies of shot noise are available [60–65]. The noise measurements are technically more difficult than the conventional conductance examinations; one of the reasons is a need of separating shot noise from background 1/*f* noise caused by fluctuations in the physical environment [66]. The shot noise is a purely nonequilibrium property that results from the fact that current is not a continuous flow but a sum of discrete pulses in time. Shot noise is a zero-frequency current noise out of equilibrium. Current and shot noise, corresponding to the average and variance of the number of electrons passing through a dot per unit time, respectively, provide different information on a transport phenomenon. Equilibrium (thermal) fluctuations can be related via the fluctuation–dissipation theorem to the linear conductance; thus, equilibrium noise does not carry extra information other than this from conductance [67]. Coulomb repulsion between electrons and their fermionic nature can regulate their motion, an effect that cannot be deduced from the time-averaged DC measurements, but may be detected in the shot noise reduction or enhancement in respect to the Poissonian value for noninteracting carriers [66,68–71]. The increasing interest in shot noise is also dictated by the possibility of extracting information about the specific, most fundamental form of correlations, namely, entanglement [72–75]. There are several theoretical proposals to form and detect entanglement in solid-state devices by means of shot noise measurements [76]. Shot noise studies are crucial for quantum computing. Shot noise, characterized by uncertainty in particle arrival times, can disrupt the superposition of states and entanglement, leading to information loss and decoherence.

The considerations made in this work are in the spirit of the effective Fermi liquid (FL) theory [77–86]. One can analyze the low-energy behavior of a multilevel interacting quantum dot (N-QD) in terms of quasiparticles and their weak residual interactions. The Kondo many-body singlet is described by us in the extended slave boson approach using the Kotliar and Ruckenstein representation (SBMFA) [87–88]. In the SBMFA, the effective non-interacting quasiparticles scatter elastically on the Kondo resonance. SBMFA correctly describes spin or pseudospin fluctuations in the unitary regime. In the large, infinite-*N* limit, this description of the SU(*N*) Kondo effect is exact. For finite *N*, however, apart from elastic scattering of quasiparticles described by energy-dependent phase shift, one must also deal with two particle scattering off the singlet. At finite degeneracy, fluctuations of slave bosons about their mean-field coherent states determine the size of current fluctuations. Motions of the quasiparticles generate fluctuations that create interactions between quasiparticles [89]. The FL parameters can be expressed in terms of linear and nonlinear susceptibilities [82]. We compute the general susceptibilities and three-body correlation functions in terms of quasiparticle Green’s functions determined with respect to the SBMFA equilibrium ground state; using them, we specify the effective interactions between quasiparticles, which in turn allows us to calculate the higher-order FL corrections to conductance, susceptibilities, and shot noise. Linear shot noise is essential around equilibrium, and it reflects the partition of the scattered particles. The meaning of this contribution to noise is consistent with Landauer–Büttiker theory. Out-of-equilibrium interaction between quasiparticles shows up, and noise is nonlinear and strongly enhanced. This is caused by two-particle and three-particle scattering events and the accompanying backscattering processes [85].

In the present paper, we show that SBMFA, which, in the strong coupling limit, satisfactorily describes strong correlations, can be used for a discussion of linear conductance and partition noise. This approach, however, as the mean-field approximation uses the picture of independent, dressed quasiparticles, neglects fluctuations. Therefore, this method cannot be used for analysis of nonlinear noise and non-diagonal susceptibilities. One way to solve this problem is to perform tedious calculations of fluctuations of boson fields around the saddle point [89–90]. The role of interaction beyond mean-field approximation can be also included by a simple, intuitive analysis based on FL theory. Following earlier reports, the needed FL coefficients can be found perturbatively [91], and they are expressed by susceptibilities. In our proposition, the diagonal susceptibilities are found directly within the SBMFA formalism; for the calculation of non-diagonal correlators occurring for interacting systems, we use a simple extrapolation of the Wilson coefficient from the case of an isolated system to a system weakly coupled to electrodes. This allows us to determine the FL free energy and, hence, the thermodynamics and nonlinear transport properties of the system.

Let us now give an overview of the most important topics of this article. The paper comprehensively analyzes the linear and nonlinear transport and thermodynamic properties of Kondo systems with SU(*N*) symmetries up to *N* = 6 with emphasis on the discussions of the shot noise. Particularly innovative are the results for systems with SU(5) and SU(6) symmetries, which have not been discussed in detail so far. Apart from numerical results for Kondo temperatures, we also derived a simple approximate analytical SBMFA formula for *T*_K_. Characterizing the used scheme, the presented study is an analysis based on FL theory with quasiparticles defined within SBMFA. To extend the calculations for high voltages, we went beyond MFA description of slave bosons and determined the auxiliary bosons in a self-consistent procedure including FL corrections. We illustrated screening of generalized SU(*N*) spins by presenting temperature dependencies of susceptibilities and entropies. Discussing nonlinear shot noise, we have shown for which occupancies two-particle correlators, and for which three-particle correlators, are dominant and how this is related to the symmetry of the correlation functions.

The remainder of the paper is organized as follows: In the next chapter, we first introduce the model and outline the many-body formalism we use, that is, the slave boson technique. Next, we present the expressions for the susceptibilities, linear conductance, and shot noise, and we briefly explain the microscopic basis of the FL theory with quasiparticles defined in the framework of SBMFA. This allows us in the next step to present and justify the formulas for the nonlinear conductance and noise. The next chapter is devoted to the numerical results and their analysis. We first discuss conductance, Wilson’s ratios, shot noise, Fano factors, and Kondo temperatures of SU(*N*) systems. We also show susceptibilities and entropies of the N-QD linked with electrodes and compare them with the case of an isolated multiorbital dot. Finally, we give conclusions and some final remarks. We complete the text with two appendices containing an approximate analytical SBMFA expression for the Kondo temperature in SU(*N*) systems and formulas for the susceptibilities and Wilson coefficients of an isolated N-QD.

## Formulation

### Model

We consider a N-QD coupled to electrodes, described by the *N*-level Anderson Hamiltonian:


[1]

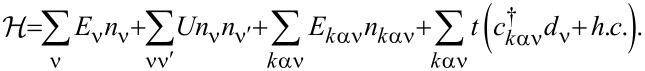



The first term represents the orbital energies *E*_ν_ = *E*_d_ (ν = 1, 2, …, *N*), the second, parameterized by *U*, describes Coulomb interactions, and the last two terms describe electrons in the electrodes and their tunneling to the dot (*t*). The Hamiltonian (Equation 1) also describes a capacitively coupled *N*-dot structure with dots connected to the separate leads. The occupation number operators of the spin-orbital and of the Fermi sea in the left (right) leads are given by 
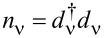
 and 
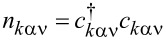
, respectively. We assume the coupling strength to the electrodes with the rectangular density of states 1/2*W* (*W* denoting the electron bandwidth of electrodes in the wide-band approximation, where the leads are represented by a flat density of states). Γ = π*t*^2^/2*W* is the coupling strength to leads. In the calculations, we use the natural units setting ℏ = *k*_B_ = *e* = *g* = |ν_B_| = 1. We also take *W*/50 as the energy unit. Although numerical calculations presented in this article only concern fully symmetric cases (degenerate levels), the presented introductory formulas are more general and refer also to non-degenerate levels. Our next publication will be devoted to changes of the shot noise along symmetry crossover [92]. In the following, we analyze strongly correlated states of the N-QD characterized by SU(*N*) symmetry, *N* = 2, 3, 4, 5, 6. In the absence of magnetic fields, for even values of *N*, degeneracy applies to both orbital (*i*) and spin (σ) degrees of freedom, and labeling ν can be understood as ν = *i*σ.

**Figure 1:**
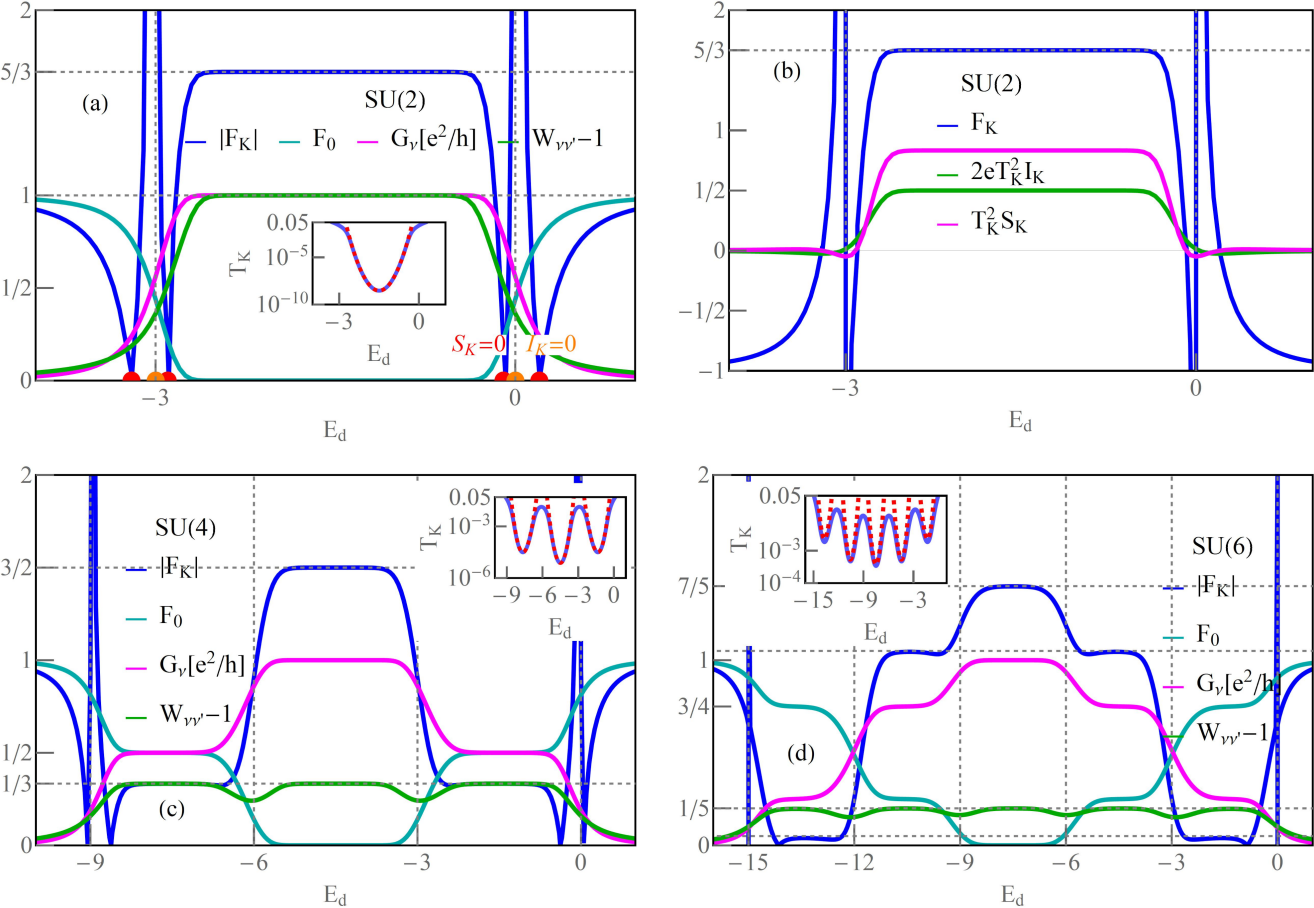
Kondo effect with even symmetry SU(*N* = 2, 4, 6): (a, c, d) linear and nonlinear Fano factors *F*_0(K)_, single-channel quantum conductance *G*_ν_, and Wilson ratio *W*_νν'_ − 1 as a function of dot energy *E*_d_ for *N* = 2, 4, 6. (b) Gate-dependent *F*_K_ compared with nonlinear current and shot noise, rescaled by the square of the Kondo temperature. The insets show numerical and analytical approximation of *T*_K_ (blue and red dashed lines) (*U* = 3, Γ = 0.025, *T* = 0, energies are given in units of *W*/50).

To analyze correlation effects, we use the finite-*U* slave boson mean-field approach of Kotliar and Ruckenstein [87]. In this approximation, the effect of Coulomb interactions is effectively replaced by interaction of quasiparticles with auxiliary bosons, which project the state space onto subspaces of different occupation numbers. The mean-field approach is correct for describing spin and orbital fluctuations in the unitary regime, and it leads to a local FL behavior at zero temperature. As an example, we show the MFA Hamiltonian (Equation 2) describing noninteracting quasiparticles in boson fields for the case of the highest of the described symmetries, that is, SU(6):


[2]

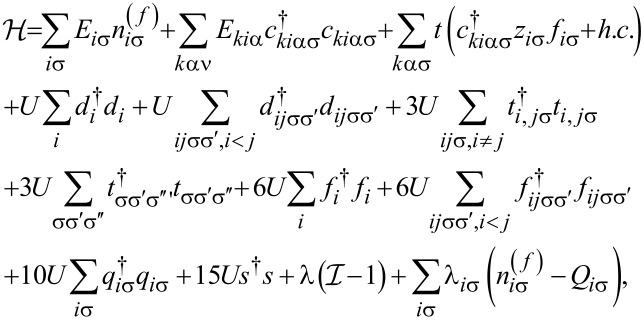



where 
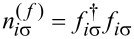
 is the pseudofermion occupation operator, {*e*, *p*, *d*, *t*, *f*, *q*, *s*} denote double, triple, quadruple, and quintuple boson fields, respectively, and λ, λ*_i_*_σ_ are Lagrange multipliers introduced to eliminate unphysical states (details of the method are presented in Appendix A). The pole of the retarded Green’s function 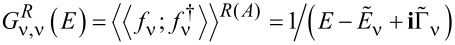 in channel ν determine position 
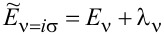
 and width of the quasiparticle resonance 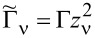. The corresponding characteristic resonance temperature is 
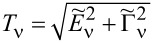
. For fully symmetric systems, there is a single resonance line, *T*_ν_ for all orbitals are equal, and *T*_K_ = *T*_ν_ is the Kondo temperature. In addition to our numerical SBMFA estimations of the Kondo temperature *T*_ν_, we also use a simple, derived by us, SBMFA approximation of *T*_K_ presented below. The derivation of Equation 3 is given in Appendix A. Equation 3 well reproduces the numerically calculated Kondo temperatures and has the advantage that it is expressed by the bare parameters of the Anderson model (insets below in Figure 1 and Figure 3). For this reason, it gives more intuitive insight into factors determining the Kondo temperature:


[3]

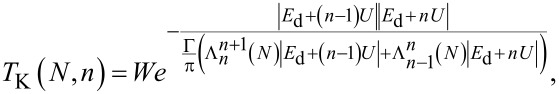



where *n* denotes the total filling of the dot; the coefficients 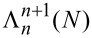 and 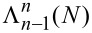 are given in Appendix A.

### Thermodynamic and transport properties: SBMFA and Fermi liquid approach

In the following, we present, based on the Fermi liquid concept, formulas for basic thermodynamic and transport quantities of the N-QD. Fermi liquid theory is used to describe the behavior of interacting fermions at low temperatures. The starting point is a non-interacting Fermi gas. We keep the considerations at a microscopic level and assume that the noninteracting quasiparticles are defined within the SBMFA formalism. Elastic scattering off the many-body Kondo singlet and weak interaction of quasiparticles induced by polarization of the spin singlet determine the higher-order corrections to physical quantities. In the first step, let us write formulas for thermodynamic and transport quantities in the non-interacting quasiparticle picture.

The zero-temperature current *I* and the shot noise can be expressed by the transmission 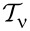 in the following form:


[4]

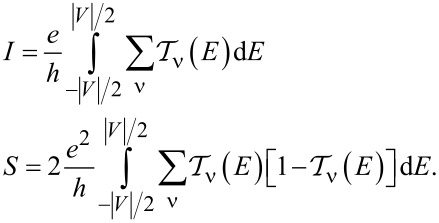



According to the Landauer–Bütikker approach [53,66–67], the low bias current *I*_0_ = *G*_0_*V* and shot noise *S*_0_ = *A*_0_*V* can be expressed in the limit of zero temperature in terms of transmission (reflection) probabilities at the Fermi energy alone. The factors (1 − 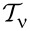) describe reduction of noise with respect to Poisson noise. The transmission 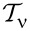 is determined by the phase shift 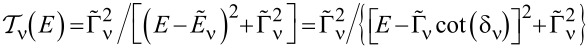 consequently, the linear conductance and linear noise ratio are given by:


[5]

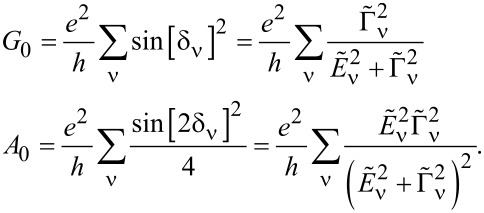



The linear zero-temperature Fano factor *F*_0_ reads:


[6]

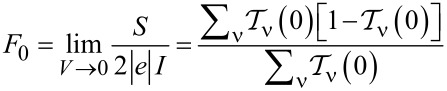



for the fully degenerate case, where all single-channel transmissions are equal, it is simplified to 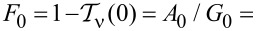 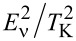. The above contribution to the noise is called partition noise because it reflects fluctuations related to partition of the scattered particles. There are no fluctuations in the number of carriers at zero temperature; shot noise reflects the character of transmission described by probabilities. Formally, the shot noise formula (Equation 4) is analogous to the free electron formula, but one has to keep in mind that, in some way, part of the electron correlations is already included in this simple approach by renormalizing the SBMFA parameters, and it properly captures the limit *V* → 0. For example, it correctly gives complete suppression of the shot noise for SU(2) Kondo systems and *S*_0_ = 2(*e*^2^/*h*) for SU(4) [62]. Linear noise is a direct signature of the symmetry class because, for each symmetry, a different number of channels are involved in transport. In the general case (finite temperature and voltage), it is necessary to take into account noise corrections beyond MFA. One needs to either consider fluctuations of slave bosons and pseudofermion operators or to find higher-order corrections to current and shot noise in the framework of the FL theory analyzing the elastic and inelastic scattering of quasiparticles. The latter approach is the path we follow in this article. FL theory applies to the low-energy properties of quasiparticles. The great advantage of this theory is that it can be also applied to nonequilibrium processes. In the Kondo problem of interest here, quasiparticles are scattered elastically by the singlet. They also interact through polarization of the singlet. The FL energy functional, which is biquadratic with respect to quasiparticle creation/annihilation operators (bilinear with respect to quasiparticle fluctuations), is parameterized by four coefficients, namely, the two postulated by Nozières [78], which modify the excitation energy in the first order and parameters of second order necessary for description of nonequilibrium quantities such as nonlinear current and noise [91,93]. Their gate-, field-, or temperature-dependencies are mostly quadratic. Many recent works on Kondo systems [94] emphasize the importance of residual interactions in current fluctuations. As shown, for example, in [82,95], all four FL parameters can be expressed, using the Friedel sum rule, by zero-temperature susceptibilities and their derivatives with respect to the local level positions. Static two-body susceptibilities 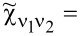 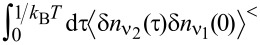 and three-body correlation functions 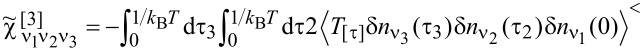 are expressed through derivatives of the free energy with respect to site energies. δ*n*_ν_ denotes deviations from the ground-state distribution, δ*n*_ν_ ≡ *n*_ν_ −⟨*n*_ν_(0)⟩. In the mean-field approximation, the free energy corresponding to the Hamiltonian in Equation 2 is a sum of slave boson free energy 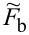, and fermionic contribution 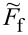 (
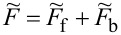
). The diagonal fermionic susceptibilities at low temperatures 
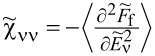
, 
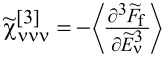
 are given by Equation 7 and Equation 8.

Commonly used quantities serving as a measure of relative strength of correlations are the Wilson ratios *W*_νν'_, defined by the proportions of the off-diagonal component of susceptibility to the diagonal component [80,82]:


[9]

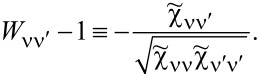



Most frequently cited in the literature, the spin Wilson ratio [80] is defined as the ratio of the spin susceptibility χ_(_*_s_*_)_ and the linear specific heat coefficient γ_(0)_ and can be written as:


[10]

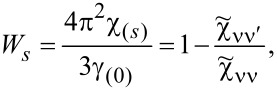



where 
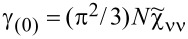
 is the linear specific heat coefficient [15]. For the fully symmetric case SU(*N*), the linear susceptibility has only two independent, generally different, components, that is, diagonal and off-diagonal. All diagonal elements 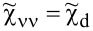 are equal and, similarly, all off-diagonal elements 
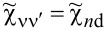
 (ν ≠ ν’). Consistently, the Wilson ratio is a single number in this case. Spin χ_(_*_s_*_)_ and charge χ_(_*_c_*_)_ susceptibilities can be written as:


[11]

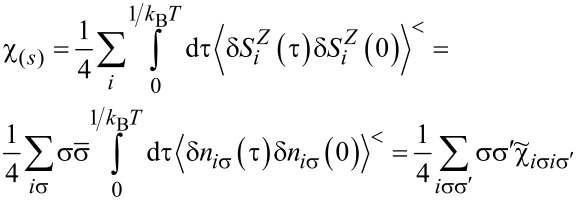




[12]

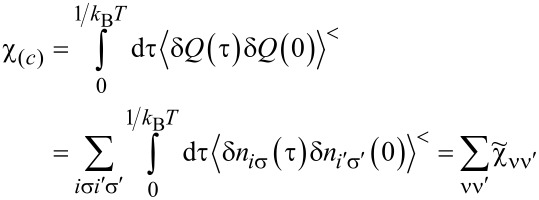



Assuming that the Wilson ratio is known and using the correlator equations (Equation 13, Equation 14), we can write the spin and charge susceptibilities for SU(*N*) system in the form:


[15]

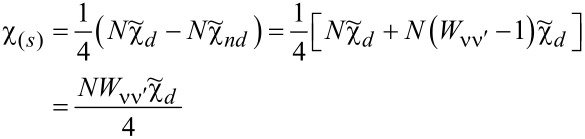




[16]

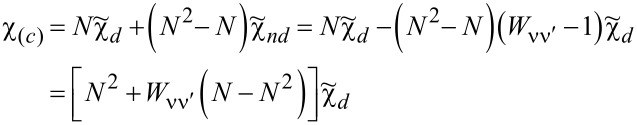



Typically, due to residual interactions, the Wilson ratio increases, but, in general, it depends on the type and strength of the interactions and the resulting many-body phenomena [15]. One of the possible reasons for the weakening of the Wilson ratio may be strong charge fluctuations. In the case discussed here (SU(*N*) Kondo states), values of *W*_νν'_ are larger than one; hence, spin susceptibilities are enhanced by residual interactions, whereas charge susceptibility is suppressed. The characteristic temperature T^*^ in which Kondo correlations become visible in susceptibilities is defined as 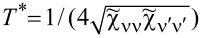 and can be considered as the Kondo temperature determined from susceptibility. For the special case of SU(2) symmetry, the Kondo susceptibility expressed by the characteristic temperature T^*^ is 
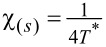
 [96]. The off-diagonal linear susceptibilities χ_νν'_ and nonlinear susceptibilities 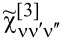 can be expressed using only Wilson coefficients and diagonal elements as:


[17]

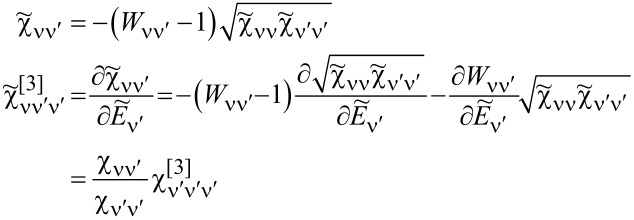



Another thermodynamic quantity revealing quenching of local moment is the entropy of an SU(*N*) Kondo dot, which we can generally express in the form:


[18]

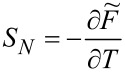



where 
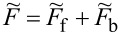
. For *T* ≪ *T*_K_, *S_N_* ≈ 0, which indicates transition to the SU(*N*) Kondo singlet. An increase in temperature leads to a saturation of the entropy at a value characteristic of the local moment where the screening effect is removed and the entropy reaches a value of *S_N_* = *k*_B_ln[*N*!/(*n*!(*N* − *n*)!)]. The essence of the Kondo effect is the formation of a singlet of a localized electron with conduction electrons. In the case of SU(2) symmetry, the screened quantity is spin or orbital (charge) pseudospin characterized by two-dimensional Pauli matrices. For an SU(3) system, three possible one-electron states are labeled by flavor: up (u), down (d), and strange (s), with the terminology borrowed from quark theory [34,47]. An eight-dimensional SU(3) spin corresponds to the eight hermitian, traceless 3 × 3 generators of the SU(3) Lie group, the Gell–Mann (G-M) matrices. In the language of information theory, Pauli matrices (*N* = 2) act on qubits, Gell–Mann matrices (*N* = 3) operate on qutrits, and, for a generic *N*-level system, the generalized G-M matrices act on qudits [97]. Among the generalized SU(3) spin components, three have vector character and five are quadrupoles. Generalizing the *i*-th component of SU(*N*) spin can be defined as follows [26,37]: 
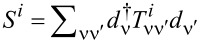
, where *T^i^* are *N* × *N* generators of the SU(*N*) group, *i* = 1, ..., *N*^2^ − 1, and ν, ν^′^ range over *N* channels. The operators *T^i^* span the full space of local physical observables. *T^i^* = (1/2)Λ*_i_*, where *i* is a generalized G-M matrix. The above choice of generators, called fundamental, is not the only one [42,44,98]; but to treat all symmetries in a unified way, we will work with this set. In the following we will use the terms SU(*N*) spin and generalized spin interchangeably. Any SU(*N*) group element can be written as:


[19]

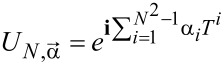



where 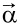 is a (*N*^2^ − 1)-dimensional vector of real parameters. The contribution of conduction electrons to the total spin has a similar form 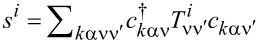, and the components of the total SU(*N*) spin *S*_tot_ are 
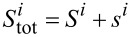
. *S*_tot_ commutes with the SU(*N*) Hamiltonian (Equation 1). In SU(*N*) symmetry, there are *N* − 1 commuting generators that play the role of *S_Z_* (represented by diagonal matrices) and *N*(*N* − 1)/2 symmetric and *N*(*N* − 1)/2 antisymmetric matrices corresponding to transverse components of *S*. The squared sum of the generators gives the quadratic Casimir operator 
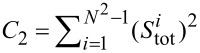
, a quantity that plays a central role in the theory of Lie groups. This operator is group-invariant and commutes with every generator of the group. The eigenvalues of Casimir elements can be used to classify irreducible representations of the group. As it is seen, *C*_2_ can be interpreted as the squared SU(*N*) spin operator. We can write the generalized SU(*N*) spin susceptibility as:


[13] $$\chi_{N}\left(T\right)=\frac{\langle S_{\text{tot}}^{2}\rangle -{\langle S_{\text{tot}}\rangle }^{2}}{T}$$

The SU(*N*) susceptibility can be expressed as:


[20]

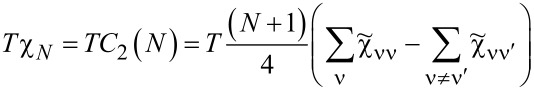



and, for *t* = 0, it is reduced to:


[14] $$T\chi_{N}=a_{N}\left(\sum_{i=\nu }Q_{i}-b_{N}\sum_{i<k}Q_{i}Q_{k}\right)=TC_{2}^{\left(0\right)}\left(N\right)$$

where *a_N_* = {3/4, 4/3, 15/8, 12/5, 35/12, …} and *b_N_* = {2, 1, 2/3, 1/2, 14/5, …}. 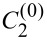 is the quadratic Casimir operator in the free pseudospin momentum limit (*t* = 0). We see that, for *Q* = 1*e*, the inter-state charge transfer correlator is *Q_ik_* = *Q_i_Q_k_* = 0. Correspondingly, successive values of *N* reach the numbers *T*χ*_N_* ≈ *a_N_*. In Figure 6 below, we present (*T*_K_ + *T*)χ*_N_*. In the low-temperature limit, *T* → 0, it reaches the characteristic value:


[21]

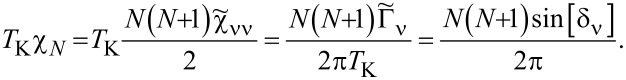



*T*_K_χ*_N_* depends on the degree of degeneration *N* and the charge (via the phase shift of δ_ν_), where (*T*_K_ + *T*)χ*_N_* = 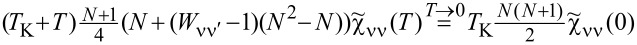. This measurable quantity provides information about the residual interaction of quasiparticles with pseudospin. At high temperatures, (*T*_K_ + *T*)χ*_N_* → *T*χ*_N_* and describes the square of the generalized spin.

### Nonlinear current and nonlinear noise

As we have mentioned in the previous subsection, linear noise (*eV* ≪ *T*_K_) is completely described by non-interacting quasiparticles. At higher voltages, some nonlinearity in the noise also appears due to nonlinear conductance, which is a consequence of the strong energy dependence of transmission. However, residual interactions play the decisive role in the enhancement of current fluctuations. Scattering of quasiparticles is characterized by an effective charge e^*^ different from the electron charge *e*. This is a consequence of the simultaneous backscattering of one and two quasiparticles [93]. The noise referring to a fractional effective charge is called fractional shot noise. The enhancement value is closely related to the Wilson ratio and is universal for the Fermi liquid in the Kondo regime as it depends only on the symmetry group of the system [68]. To analyze higher-order corrections to the shot noise generated by interactions, one has to supplement the mean-field slave boson free energy (Equation 22) by at least bilinear terms with respect to population fluctuations. Noziéres first formulated a microscopic Fermi liquid theory for the Kondo model [84]. He derived Fermi liquid coefficients (i.e., α_1_*_,_*_ν_ and φ_1_*_,_*_νν'_) analyzing ff-singlet quasiparticle scattering and expanding the corresponding phase shifts (δ_ν_(*E*,*n*_ν_)) in leading order in energy and deviations of the quasiparticle distribution function from its ground state. To describe nonequilibrium properties away from particle hole symmetry points apart from Noziéres coefficients, which are first order in the excitation energy, also additional second-order coefficients are required. A clear presentation of a microscopic scheme for determining Fermi liquid coefficients and nonlinear noise is presented, for example, in [81–82,84]. The current–current correlation function, which is the main subject of this paper, 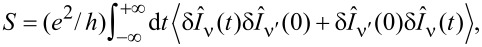 where 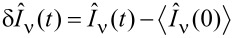, depends on the collision term of two quasiparticles, which is described by the Keldysh vertex corrections. Oguri and colleagues [82] calculated the vertex functions up to the linear order with respect to the bias voltage *V*, the temperature *T*, and the energy *E*. The Keldysh Green’s functions were expanded up to terms of order *V*^2^, *T*^2^, and *E*^2^. Fermi liquid coefficients were deduced from derivatives of self-energy and vertex functions using the Ward identities [82,99], which relate these quantities. It was shown by the authors, that the free energy coefficients can only be expressed by using static linear 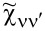 and nonlinear 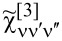 correlation functions. The derived formula for the free energy is given by [81]:


[23]

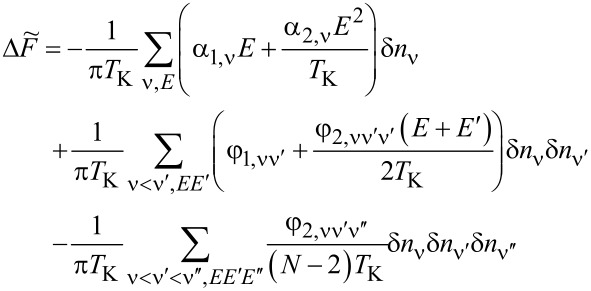



where 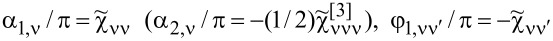 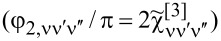 are the FL coefficients [84]. Equation 23 includes all first- and second-order terms in the low-energy coupling strength 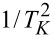. The first term of Equation 23 describes elastic scattering off a Kondo singlet and is reflected in the energy (spin) dependence of the phase shift. The second interaction term is related to the inelastic scattering and quasiparticle interaction. The last term of the expansion (Equation 23) can be formally interpreted as effective three-body interaction. The three-body contribution is essential outside the electron–hole symmetry point (*n* = *N*/2) and for systems with broken symmetry. Single-particle energies of quasiparticles are measured with respect to the Fermi energy, δ*n*_ν_ denote deviations from the ground-state distribution, and FL coefficients are expressed by static, in our considerations SBMFA, susceptibilities. The part of the bilinear correction, if included in the Hartree approximation, only modifies the elastic scattering contribution, while the rest describes inelastic collisions [84]. In general, for specifying FL parameters both diagonal and off-diagonal elements of susceptibility are necessary. Off-diagonal susceptibility appears only if residual interactions are present in the system. Our calculation methodology is the following: We directly use the formula in Equation 23 and determine the FL coefficients using diagonal correlators calculated in the SBMFA scheme. Off-diagonal correlators, which are the result of residual interactions, are also required. They do not appear in the mean-field formalism, but can be determined using diagonal correlators and the Wilson coefficient. We introduce a simple extrapolation of the Wilson coefficient from the solvable case of an isolated system to a weakly coupled system with electrodes. When the Wilson coefficients are known, the off-diagonal correlators can be directly expressed in terms of the diagonal terms in Equation 9 and Equation 17. All susceptibility elements are easily found for interacting dots disconnected from electrodes (*t* = 0). The spectrum of the isolated N-QD can be calculated exactly, and the corresponding exact free energy can be found. Susceptibilities are then calculated in the standard way differentiating the free energy over the appropriate energies. Consequently, one finds also Wilson ratios 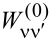 for the isolated N-QD. They can be can be expressed by quasiparticle occupations as follows (Appendix C):


[24] $$W_{\nu \nu^{\prime }}^{\left(0\right)}-1=\frac{n_{\nu }n_{\nu }-n_{\nu \nu^{\prime }}}{\sqrt{\delta n_{\nu }^{2}\delta n_{\nu^{\prime }}^{2}}}=\frac{Q_{\nu }Q_{\nu^{\prime }}-Q_{\nu \nu^{\prime }}}{\sqrt{Q_{\nu }\left(I-Q_{\nu }\right)Q_{\nu^{\prime }}\left(I-Q_{\nu^{\prime }}\right)}}$$

For simplicity of calculation, we introduce the following approximation, which will help us easily find approximate values of Wilson ratios and, consequently, also non-diagonal susceptibility elements for the system linked with the electrodes. Considering the low value of the quasiparticle renormalization weight *z*_ν_ in the SBMFA approach, it is reasonable to assume that extrapolation of Equation 24 to the range of small values of hopping parameter *t* should be a good approximation for Wilson ratio and static correlators. Using Equation 9 for the N-QD connected to electrodes, the assumed quasiparticle occupation should correspond to this case (*t* ≠ 0). This simplification allows us to calculate all components of susceptibility avoiding at this stage the use of the tedious advanced many-body approaches. One can easily improve this approximation by introducing a self-consistency procedure for susceptibility and Wilson coefficients; however, this seems unnecessary because already this simple approximation proposed above gives satisfactory results. It well reproduces NRG calculations of two- and three-body quantities [83].

Before commenting on the contribution to the noise from interactions, it is worth mentioning that, for higher voltages, part of nonlinearities also appear without interactions due to nonlinear conductance. To analyze higher-order noise corrections generated by interactions, one has to consider the free energy interaction term 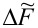. Parts of it (terms α_1(2)_*_,_*_ν_) describe elastic scattering off the Kondo singlet and are reflected in the energy (spin) dependence of the phase shift. Interaction terms (φ_1(2)_*_,_*_νν'_) are the source of inelastic scattering, but, as we mentioned earlier, Hartree terms stemming from this contribution also formally contribute to elastic scattering, and they give the mean-field energy shift of the Kondo resonance. A full account of the consequences of 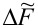 on the conductance and shot noise requires the use of the Keldysh formalism. Collision terms of two quasiparticles are microscopically described by the Keldysh vertex corrections. The Keldysh Green’s functions are expanded up to the terms of order (*eV*)^3^ to include multiple collision processes contributing to the noise; the vertex function has to be determined up to linear order. Leaving all the details, we write, according to [82], the final expressions (Equation 25 and Equation 26) for the current and shot noise given with accuracy of the order of (*eV*)^2^ and (*eV*)^3^, respectively, and expressed by equilibrium susceptibilities. To maintain the accuracy of (1/*T*_K_)^2^, both two- and three-body correlations contributing to nonlinear current and noise have to be taken into account. The details of derivation can be found in [82]:


[25] $$I=I_{0}+I_{\text{K}}=G_{0}V+\sum_{\nu }c_{V,\nu }V^{3}$$

[26] $$S=S_{0}+S_{\text{K}}=A_{0}V+\sum_{\nu }c_{S,\nu }V^{3}$$

Current and shot noise refer to nonequilibrium, where the time is broken; therefore, in Equation 25 and Equation 26, only odd terms of *V* occur. The formulas for linear current (*I*_0_) and partition noise (*S*_0_) have been given earlier, and the nonlinear coefficients *c_V,_*_ν_ and *c_S,_*_ν_, derived in [82], are presented in Appendix D. The generalized Fano factor *F*_K_, which describes the relation between the nonlinear current and current noise is [82]:


[27] $$F_{\text{K}}=\frac{{\text{d}}^{2}S/\text{d}V^{2}}{2\left|e\right|{\text{d}}^{2}I/\text{d}V^{2}}=\frac{S-S_{0}}{2\left|e\right|\left(I-I_{0}\right)}=\frac{S_{\text{K}}}{2\left|e\right|I_{\text{K}}}=\frac{\sum_{\nu }c_{S,\nu }}{\sum_{\nu }c_{V,\nu }}.$$

## Results

We present numerical results for the *N*-degenerate quantum dot described by the *N*-level Anderson model. Our study examines the impact of correlations on transport and thermodynamic properties of an SU(*N*) Kondo dot with special focus on the shot noise. As mentioned in the Introduction, the following considerations are not limited to the multilevel dot, but they can as well be addressed to multiple dots and various other strongly correlated systems described by the Hamiltonian in Equation 1 and characterized by special unitary symmetries. To discuss the correlations, we use the extended Kotliar–Ruckenstein slave boson formalism in the mean-field approximation [10,87–88]. The calculations were carried out assuming Coulomb parameter *U* = 3 and coupling to the leads Γ = 0.025. Before we get to the results, let us mention that the assumption of weak electrode coupling (*U*/Γ = 120) ensures achieving Kondo’s unitary limits in calculations for all the analyzed symmetries, which manifests in the occurrence of flat plateaus of conductance with unitary values and, correspondingly, also flat lines of Fano coefficients in Kondo regimes and convergence of Wilson ratios to the values 1/(*N* − 1). The assumed ratio of coupling strength and Coulomb interaction parameter are within the range of experimental data. The value of *U* can be inferred from the size of Coulomb diamonds. For semiconducting carbon nanotubes, the charging energy is of the order of tens of millielectronvolts [7,29] and, for QDs in a 2DEG, *U* = 1–4 meV [6,100–101]. Γ gives us a quality of contacts and is of the order of millielectronvolts or even its fractions [7,100]. In the following figures, we present Kondo temperatures, conductances, Wilson ratios, Fano factors, susceptibilities, and entropies as functions of current or gate voltage.

### Even symmetries of Kondo states

Figure 1 concerns symmetries of even rank, SU(*N* = 2, 4, 6). Some aspects regarding the first two symmetries have been discussed earlier, for example, in [7–8,24,28–29,62,94,102]; here, we present these results mainly for the sake of completeness of the discussion and for comparative purposes. The chosen ratio *U*/Γ = 120 ensures the strong correlation limit of the analyzed systems. As mentioned earlier, for Γ = 1–5 meV, the value of the Coulomb parameter *U* (*U* = 5–30 meV) corresponds to carbon nanotubes [7,29], and *U* = 1.5–4 meV refers to semiconducting quantum dot systems with Γ = 0.1–0.4 meV [5–6]. The calculated SU(2) conductance per single channel visible in Figure 1a for *n* = 1 approaches unitary Kondo limit *G*_ν_ = *e*^2^/*h*, which agrees with the predicted value based on the Friedel sum rule linking conductance with the phase shift, or equivalently with the charge on the dot. The phase shift δ_ν_ = π/2 determines the total conductance 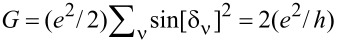. The Fano factor in the linear range reduces to zero (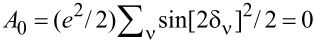). SU(2) Kondo resonance is pinned at the Fermi level (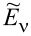 ≈ 0) and, consequently, transmission 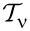 → 1; thus, an absence of partition noise is observed. As can be seen in Figure 1a, the general dependence of the Fano factor *F*_0_ on the gate potential resembles an upside-down curve of the conductance. Although partition noise for an SU(2) Kondo device is equal to zero (*F*_0_ = 0), for finite bias voltage, transport in this state is noisy due to quasiparticle interactions (Figure 1a, Figure 4a). For *N* = 4 (Figure 1c), three plateaus of conductance are observed, lower *G*_ν_ = (1/2)(*e*^2^/*h*) for odd Kondo effects (*n* = 1, 3) (δ_ν_ = π/4, 3π/4) and higher for even Kondo effect *G*_ν_ = *e*^2^/*h* (*n* = 2, δ_ν_ = π/2). For half-filling, six local two-particle states participate in the formation of the Kondo resonance. In general, for a given degeneracy *N* and occupancy *n*, the number of *n*-particle local states involved in Kondo fluctuations is *N*!/(*n*!(*N* − *n*)!). At quarter filling, Kondo resonance is shifted from the Fermi energy and transmission 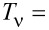 1/2, which creates a strong partition noise. For *n* = 2, perfect conductance appears with the consequent absence of partition noise. Two characteristic values of δ_ν_ for SU(4) symmetry are reflected in two values of plateaus in the linear Fano factors *F*_0_ presented in Figure 1c. In the regions of odd occupancies *F*_0_ = 1/2, whereas for even occupancies *F*_0_ vanishes (noiseless, ballistic transport for ultra-low voltages). The noise measurements carried out in single-wall carbon nanotube-based quantum dots (SU(4)) by Delattre et al. [62] confirm this result and prove that *F*_0_ calculated by SBMFA, describing shot noise of renormalized independent particles, accurately estimates the noise in the low-temperature, low-voltage regime. The earlier calculations of partition noise for SU(4) Kondo systems can be found in [82–85,94–95]. For SU(6) symmetry (Figure 1d), Kondo effects occur for regions characterized by occupancies *n* = 1, 2, 3, 4, 5. At half-filling, the resonance is again centered at the Fermi level, and twenty local three-particle states participate in Kondo fluctuations. Similarly to the earlier discussed symmetries, for *n* = 3, fully transmitting, noiseless channels occur at low voltages (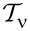 → 1, *A*_0_ → 0, Equation 6). Conductances of SU(6) Kondo states per single quantum channel for different gate voltages are *G*_ν_ = (*e*^2^/*h*)sin[δ_ν_]^2^ = 1/4(*e*^2^/*h*), δ_ν_ = π/6 for *n* = 1, 5, *G*_ν_ = 3/4(*e*^2^/*h*), δ_ν_ = π/3 for *n* = 2, 4, and *G*_ν_ = *e*^2^/*h*, δ_ν_ = π/2 for *n* = 3. Outside the half-filling region, partition noise of SU(6) Kondo states is finite. Insets of Figure 1a,c,d present Kondo temperatures 
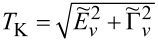
. For each degeneracy *N*, the lowest value of Kondo temperature occur at half-filling. It is related to the fact that, for *n* = *N*/2, the electrode channels link with the largest number of local states *M* (*M > N*). This indicates that the number of states participating in fluctuations or the number of relevant bosonic fields decisively influence on the values of coefficients 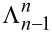 (Equation 28) and 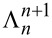 (Equation 29), and for half-filling it is maximal. The red dashed lines shown in the insets correspond to the analytical slave boson formula (Equation 3) expressing the Kondo temperature through the bare parameters of the Anderson model. The solid blue lines present numerical calculations based on the formula 
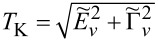
, which is similar to Equation 30. The agreement of analytical solution with the numerical calculations is satisfactory. One should exclude in comparison the boundary regions between Coulomb valleys because they are poorly described within SBMFA. While the SBMFA picture of the free quasiparticles is correct near the equilibrium state, for description of the enhancement of current fluctuations beyond the equilibrium, it is necessary to take into account the residual interactions between quasiparticles responsible for the two-particle scattering processes. The importance of residual interactions is manifested in the increase of Wilson ratio in the Kondo range; for SU(2) symmetry, it reaches the value *W*_νν'_ = 2 (Figure 1a). Similarly, Wilson ratios for Kondo states in SU(4) and SU(6) symmetries are *W*_νν'_ = 4/3 (Figure 1c) [96] and *W*_νν'_ = 6/5 (Figure 1d), respectively. For the fourfold degeneracy, both spin and orbital pseudospin Wilson coefficients are equal. Wilson ratios decrease with increasing *N*, signaling the weakening of correlations. It is worth noting that, approaching the regions of occupancies *n* = 0 or *n* = *N*, *W*_νν'_ tends to one, which means that the picture of non-interacting particles gradually begins to apply there. At the borders of Coulomb valleys (mixed valence range), Wilson ratios still take significant values. This indicates that significant correlations persist also in these areas due to the proximity of strongly correlated regions. The key source of nonlinear noise is backscattering. The flow of single quasiparticles and pairs can be viewed as a current carried by two different charges *e* and 2*e*, and they are backscattered with different probabilities [93,103]. Simultaneous presence of one- and two-particle scattering can be interpreted in the language of the universal effective average charge e^*^ [93]. A direct measure of an average charge is the ratio of the shot noise and the backscattered current (nonlinear Fano factor) *F*_K_ = *S*_K_/2*eI*_K_, *F*_K_ = e^*^/*e* (fractional shot noise). At half-filling, e^*^/*e* = 5/3 for SU(2) symmetry, e^*^/*e* = 3/2 for SU(4), and e^*^/*e* = 7/4 for SU(6). The mentioned values have been confirmed in experiments for SU(2) systems in QDs [70–71,104] and in CNT QDs for SU(4) [62–63], which confirmed the necessity of taking into account in the discussion of nonlinear noise the effects of interactions. They were neglected in earlier theoretical discussions [62,105–106]. The backscattering events are reflected in the increase of mixed two- and three-body correlation functions (Equation 17) and in the consequent increase of coefficients *c_V,_*_ν_ and *c_S,_*_ν_ (Equation 31 and Equation 32), which determine nonlinear current *I*_K_ and nonlinear noise *S*_K_. With the increase of bias voltage, shot noise terms of power *V*^3^ begin to dominate over the linear contribution. The significance of three-body correlations has been confirmed experimentally in [85].

Figure 1b shows the variation of the nonlinear Fano factor *F*_K_ as a function of the atomic level of the quantum dot *E*_d_ for the Kondo state with SU(2) symmetry. In the regions of the empty state 0*e* and the fully occupied state 2*e*, the Fano factor reaches the value −1, which is related to the dominant influence of the two-body correlation functions 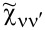 in *c_V,_*_ν_ (see Equation 31). In the region with one electron *Q* = 1*e*, *F*_K_ is 5/3, which is in agreement with literature reports [68,84,93,103–104]. In this area, 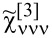 and 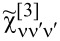 are close to zero. The three-body correlators, as the odd functions of the gate voltage, change the sign in the region with a single electron. The green and magenta lines in Figure 1b show the values of the nonlinear current normalized by the squared Kondo temperature 
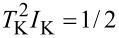
 and the shot noise 
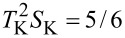
. This gives the value of the nonlinear Fano factor *F*_K_ = e^*^/*e* = 5/3. Formally, we can write the nonlinear current *I*_K_ and its fluctuating part *S*_K_ (first moment of the current correlator) as a sum of two-body and three-body contributions 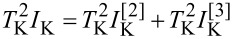 and, similarly, 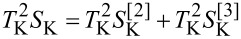. Near the triple degeneracy points (equality of SB amplitudes 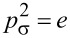 and 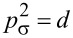), the current *I*_K_ changes its sign, and, for *I*_K_ = 0, the nonlinear Fano factor diverges *F*_K_ → ±∞. This is characteristic for the transition between the Kondo state and the Coulomb blockade regime. Around this transition region, we observe little negative nonlinear shot noise *S*_K_ *<* 0. *S*_K_ is the nonlinear contribution to the shot noise and it might be negative, but the total value *S* remains positive. In the empty region and for double occupancy *n* = 2, the nonlinear noise is positive, but small, *S*_K_ *>* 0. There are two pairs of points below and above *E*_d_ = 0 and *E*_d_ = −*U*, for which *F*_K_ is zeroing (Figure 1a,b). Similar behavior is also observed for higher symmetries (Figure 1c,d) (close to *E*_d_ = 0 and *E*_d_ = −3*U* or *E*_d_ = −5*U*). For an SU(2) dot near *E*_d_ = 0 and *E*_d_ = −*U*, also points of vanishing of current are observed (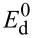) (orange dots in Figure 1a). Slightly moving from points 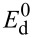 ≈ 0, −*U* current *I*_K_ changes sign in a narrow range of gate voltage due to the three-body correlations (three-body contributions) to coefficients *c_V,_*_ν_ (Figure 1b). Around the *S*_K_ zeroing points, there is also a change in the sign of the nonlinear contribution to the noise. The giant values of |*S*_K_| observed in Figure 1a,c,d can be interpreted according to the accepted terminology as hyper-Poissonian noise [84,95]. Careful insight into Figure 1 reveals that this strong enhancement of *F*_K_ is due to the occurrence of very small current in these regions. For even symmetries SU(*N* = 2, 4, 6) at half-fillings, the nonlinear noise factor *F*_K_ takes the value *F*_K_ = (8 + *N*)/(4 + *N*). The equivalent expression can be found in [83].

**Figure 2:**
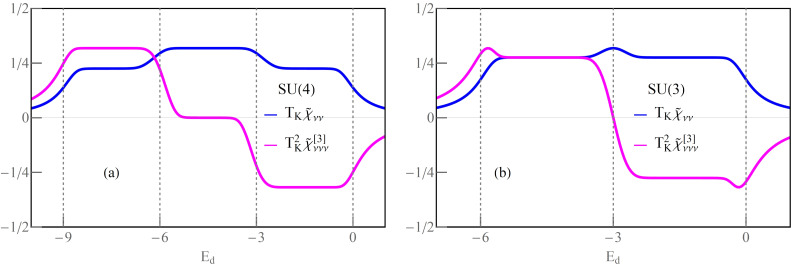
Two- and three-body correlators rescaled by *T*_K_ for SU(4) and SU(3) Kondo states (blue and magenta lines). The parameters used in calculations are the same as in Figure 1.

As mentioned earlier [68,84,93,103], three-body correlations vanish at the e–h symmetric point, and, around it, they are small and change signs. In this region, it is the two-body correlation 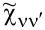 that modifies current *I*_K_ and noise *S*_K_. For *n* = 1, 3 in the SU(4) system *F*_K_ = 1/3, the noise is reduced and of particular importance there are three-body correlation functions 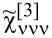 and 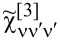 (Figure 2a). In the *Q* = 1*e*, 5*e* charge regions of the SU(6) system, the linear Fano factor is *F*_0_ = 3/4, and, for high voltages, the Fano factor is reduced to *F*_K_ = 1/20 (sub-Poissonian noise). Again responsible for this behavior are the three-particle correlations 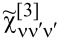 dominating in these sectors. In the charge regions *Q* = 2*e*, 3*e*, there is a reversed behavior, increase of *F*, *F*_0_ = 1/3, 0, and *F*_K_ = 21/20, 7/5, respectively, indicating that the residual quasiparticle interactions in the Kondo system cause the super-Poissonian shot noise. Since the interplay of two- and three-body correlations functions decides about the value of the nonlinear Fano factor (Equation 31–Equation 33), we present in Figure 2a,b the gate dependencies of these correlations for exemplarily chosen even SU(4) and odd SU(3) symmetries. The quantities have been rescaled by the Kondo temperature and its square, respectively (see Equation 34 and Equation 35). 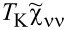 is an even function of the gate voltage, whereas 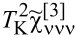 is an odd function with a negligible contribution around the e–h symmetry point. The small peaks visible on the correlators for SU(3) symmetry occur in the region of Coulomb blockade boundaries, similar peaks appear also for other odd symmetries (not presented). As can be seen in the following figures, this feature is reflected in the gate dependencies of other physical quantities for odd symmetries.

### Odd symmetries of Kondo states

**Figure 3:**
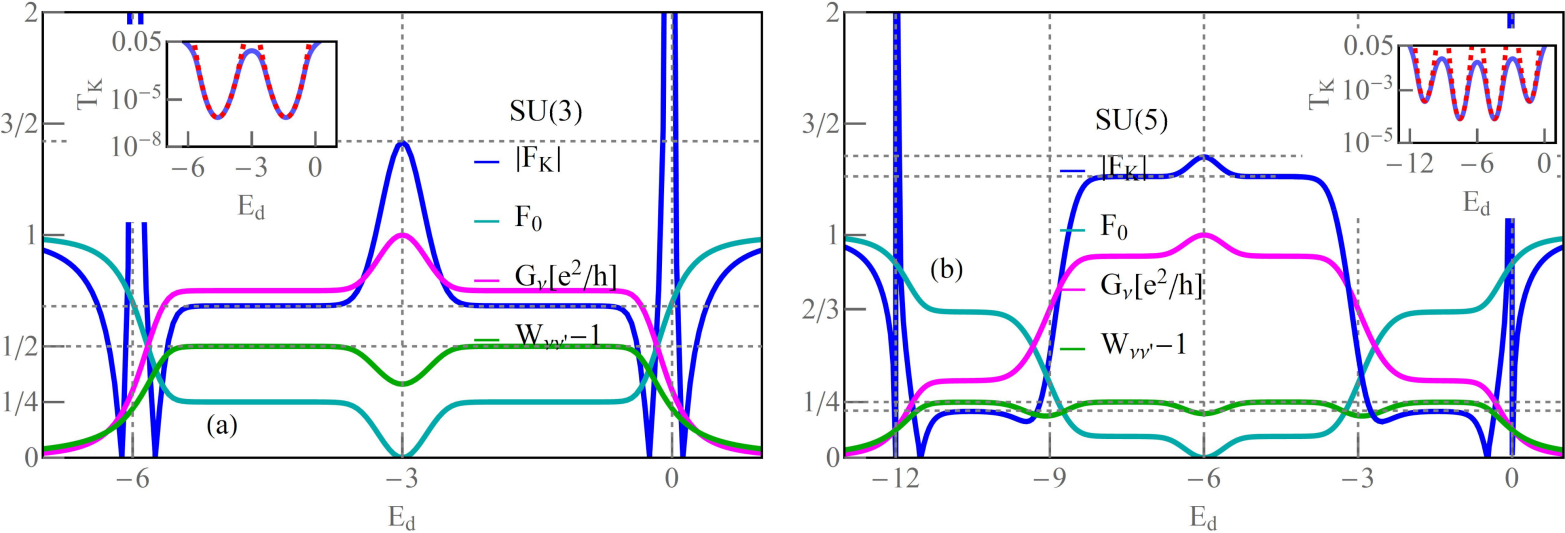
Odd SU(3) and SU(5) Kondo effect: (a,b) Gate-dependent *F*_0(K)_, *G*_ν_ and *W*_νν'_ − 1. Insets show numerical and analytical approximation of *T*_K_ (blue and red dashed lines) (*U* = 3, Γ = 0.025, *T* = 0).

Figure 3 shows results for Kondo effects with odd degeneracies (*N* = 3, 5). The e–h symmetry points in these cases correspond to non-integer occupancies (*n* = 3/2, *n* = 5/2), and high conductances at these points are associated with the charge resonances centered at the Fermi level. The dependence of conductance on the gate potential at half-filling is characterized by the occurrence of a peak instead of a plateau as in the case of Kondo resonances. In general, however, at e–h symmetric points, the linear conductance for any value of *N*, regardless of the type of resonance at the Fermi level, takes the unitary limit. For fillings (*n* = 1, ..., *N* − 1), conductance plateaus are observed, which manifest Kondo effects for these occupations. For SU(3) symmetry, the value of conductance for *n* = 1 is (3/4)(*e*^2^/*h*) and, correspondingly, the total conductance is *G* = (9/4)(*e*^2^/*h*); interestingly, this is the highest total value in the single-charge sector among SU(*N*) Kondo systems. The curves of linear Fano factors around half-fillings form deeps with *F*_0_ = 0 for e–h symmetric points; in Kondo regions *F*_0_, they take the values according to the known relationship 
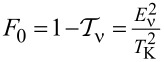
. Wilsons ratios decrease with the increase of *N* (weaker correlations) and are reduced in the areas of charge fluctuations, but, noteworthily, they still have significant values there. Apart from singularities of *F*_K_ occurring for *I*_K_ = 0, that is, points located close to empty (*n* = 0) or fully occupied regions (*n* = N), an enhancement of *F*_K_ near the e–h symmetric point is observed *F >* 1 (the super-Poissonian noise). For *N* = 3, *n ≈* 3/2 three single-electron states become degenerate with three two-electron states. The probability of the occurrence of pairs of quasiparticles is growing in this region, which is associated with an increase in the probability of backscattering of pairs. For *N* = 5, similarly as for other cases, the numerically calculated plateaus of Kondo conductance for given charge sectors agree with the formula *G*_ν_ = (*e*^2^/*h*)sin^2^[*Q*_ν_].

For integer values of charge, the Wilson ratio is *W*_νν'_ − 1 = 1/4, and, at the boundaries of charge sectors, it is *W*_νν'_ − 1 = 1/5. For occupancies *n* = 1,4, that is, for ranges close to the empty or fully occupied regions, the probability of occurrence of pairs is low, and the backscattering of pairs weakens so much that the nonlinear Fano coefficient *F*_K_ = 1/5 becomes smaller than the linear noise factor *F*_0_ = 2/3. For higher occupancies *n* = 2,3, pairs of quasiparticles are starting to play a significant role and, as we have checked numerically, both pair correlation functions 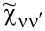 and nonlinear correlations 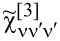 significantly contribute to the nonlinear noise; *F*_K_ in this range reaches the value *F*_K_ = 5/4 and dominates the linear noise (*F*_0_ = 1/8).

### Equilibrium versus non-equilibrium noise

**Figure 4:**
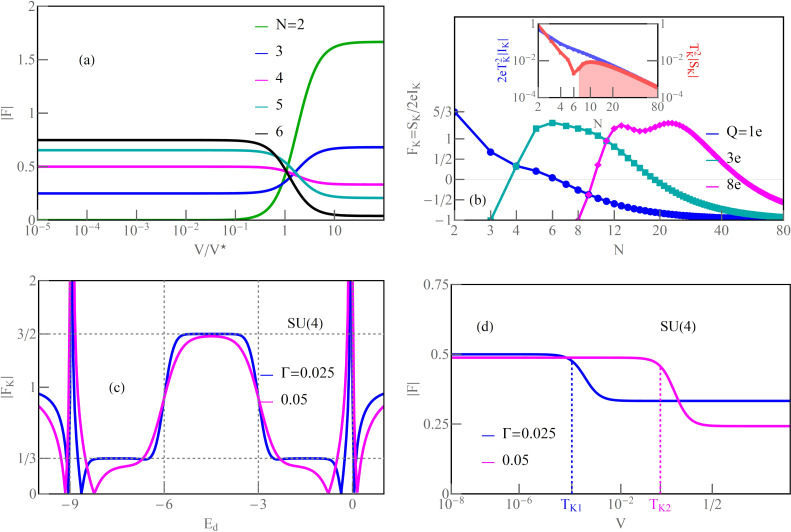
(a) Total Fano factor |*F*| versus *V*/V^*^ for SU(*N*) Kondo states (*Q* = 1*e*). (b) Nonlinear Fano factor *F*_K_ as a function of degeneracy number *N* for *Q* = 1*e*, 3*e*, 8*e* (Γ = 0.025, *V*/V^*^ = 10). The inset compares 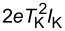 and 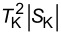 with an increase of *N* (blue and red lines, *Q* = 1*e*). The area filled in red indicates *S*_K_ *<* 0. (c,d) *F*_K_ as a function of *E*_d_ and bias-dependent |*F*| for the SU(4) Kondo state (*U* = 3, *T* = 0). *T*_K1_ and *T*_K2_ in Figure (d) represent two Kondo temperatures for *V* = 0.

Figure 4a illustrates voltage dependencies of the total Fano factor *F*(*V*/V^*^) for the exemplarily chosen filling *n* = 1 (the characteristic value V^*^ is defined in Appendix A). In the linear regime, Fano factors *F*_0_ depend only on symmetry and they increase with the increase of degeneracy. This tendency reflects the increasing shift of the Kondo resonance from the Fermi level with the growth of *N*. For *N* = 2,3, where residual interactions are stronger than for higher degeneracies, one observes the increase of *F* with voltage and its saturation for high values of *V*, *F* = 5/3, 15/22, respectively. For *N* ≥ 4, *F* at high voltages tends to very small values *F* → 1/3, 0.316, 1/20 for *N* = 4, 5, 6, respectively. These limits are solely determined by symmetry, which Equation 36 directly shows. In this equation, |*F*| depends on the degeneracy *N*, and the Wilson ratio, which is directly related to symmetry *W*_νν'_ = 1 + 1/(*N* − 1).

Figure 4b compares the dependencies of the nonlinear Fano factor on the rank of the SU(*N*) group for the selected occupancies. *F*_K_ *>* 1 indicates the dominance of bunching processes. This occurs for *Q* = *N*/2. In the systems with a larger number of electrons, the ranges in which quasiparticle grouping processes dominate (*F*_K_ *>* 1) shift to higher degenerations (*N*) (Figure 4b). For *N* = 3, the noise lowers |*S*_K_| *<* 2*e*|*I*_K_|. For *N* = 7, the influence of three-particle correlation becomes dominant 
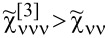
, which consequently leads to negative noise (*S*_K_ *<* 0, inset of Figure 4b). For *Q* = 3*e* and 8*e*, and degenerations *N* = 3 and *N* = 8, the nonlinear Fano factor takes the value *F*_K_ = −1, because two-particle effects in the current dominate there and *I*_K_ *<* 0. Interesting to note are the formation of a maximum for *N* = 6 (*Q* = 3*e*) and two maxima for *Q* = 8*e* (*N* = 13, 23). These effects have no fundamental cause; they occur only due to the trigonometric functional dependencies of the phase shifts 2δ_ν_ and 4δ_ν_ in the nonlinear noise coefficient (see Equation 32). The nonlinear contribution to noise (Equation 36) leads to *F*_K_ = −1 in the limit *N* → ∞ (Figure 4b).

The inset in Figure 4b shows nonlinear current and noise as functions of *N*, scaled by *T*_K_ for *Q* = 1*e*. The calculations were performed for a voltage value of *V*/V^*^ = 10. In this range, 
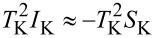
, the negative value of the noise is close to the nonlinear value of the current and, therefore, *F*_K_ = −1 (area shaded in red for *N >* = 7, inset Figure 4b). For *N* = 2, super-Poissonian processes are dominant; consequently, 
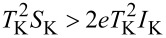
 (inset Figure 4b), when *N >* 2, there is a reversal of the trend 
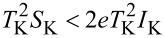
. This is due to the increase of the number of three-particle correlators, which grows with the number of quantum channels. In the case of *Q* = 1*e,* it happens for *N >* = 7.

Figure 4c,d shows the effect of the coupling strength Γ on |*F*|. An increase of Γ increases the Kondo temperature (vertical dashed lines in Figure 4d). It causes also a departure from the strictly unitary limits of the SU(4) Kondo effect, that is, from *F*_0_ = 1/2 and *F*_K_ = 1/3. The strongest modification occurs for *Q* = 1*e*, 3*e*, where the charge boundaries 1*e*/0*e* and 3*e*/4*e* are closest. The region is broadened due to the three-particle correlations. For half-filling, we observe a slight deviation from the value *F*_K_ = 3/2 due to a slight drop of the Wilson ratio *W*_νν'_ = 4/3.

**Figure 5:**
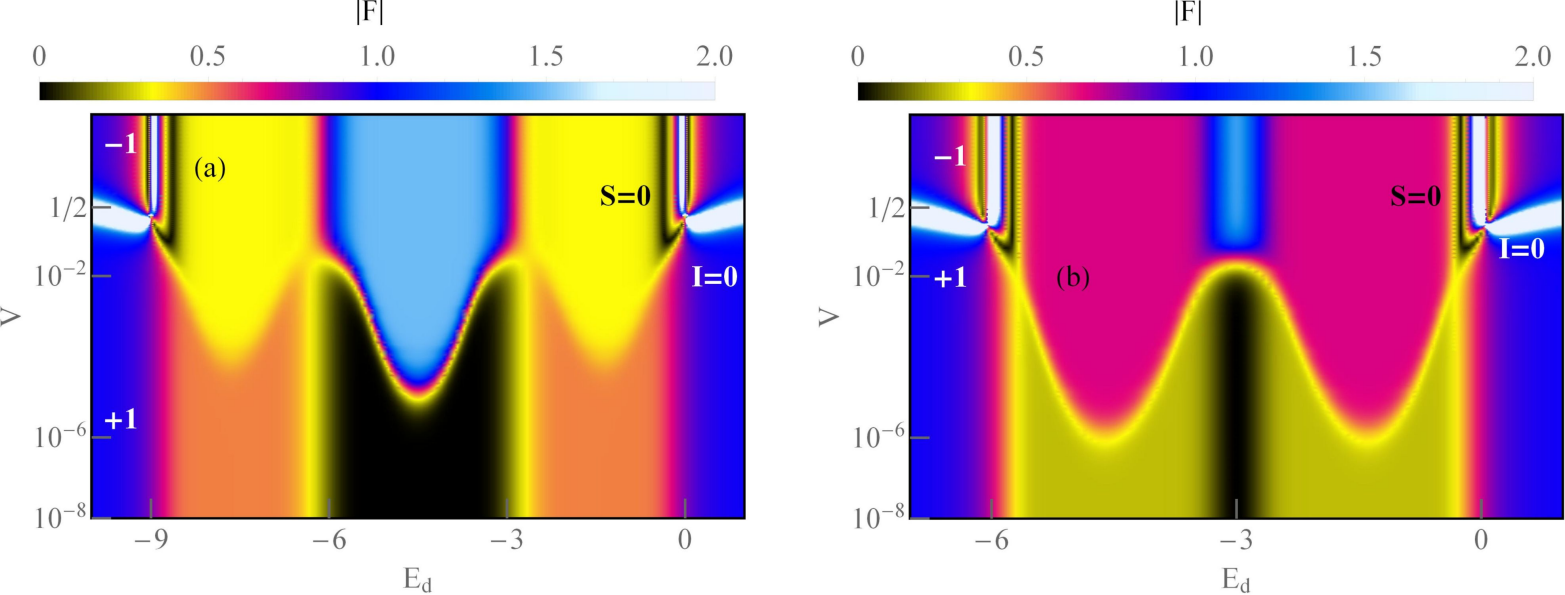
Density plots of gate-dependent total Fano factor |*F*| as function of bias voltage *V* for (a) SU(4) and (b) SU(3) Kondo states (*U* = 3, Γ = 0.025, *T* = 0). ±1 indicates the sign of the Fano factor.

The density maps in Figure 5 show the Fano factor *F* as a function of *V* and *E*_d_ for the symmetries SU(4) and SU(3). We observe an oscillating line, around *V* ≈ V^*^, separating the linear region *F*_0_ from the nonlinear Fano factor *F*_K_. This line corresponds to the characteristic voltage V^*^ = 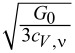 ≈ *T*_K_, where the slope saturates up to *F*_K_. For V > V^*^ the picture of non-interacting Kondo particles falls down, and nonlinear current and shot noise are significant (see Appendices A and D).

For the SU(4) Kondo state, regions 1*e* and 3*e* show the change from *F*_0_ = 1/2 (orange sector) to |*F*_K_| = 1/3 (yellow sector) (Figure 5a). In the case of half-filling *Q* = *N*/2 = 2*e*, there is a transition from |*F*| = *F*_0_ = 0 up to |*F*| = |*F*_K_| = 3/2. For SU(3), the balance of two-body (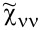) and three-body correlations (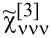) in shot noise reverses the trend, in this case *F*_0_ = 1/4 (yellow sector) and *F*_K_ ≈ 0.68 (magenta sector) (Figure 5b). This opposite behavior in the case of these two symmetries is explained by Figure 2, where we see that, for SU(4), 
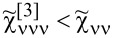
, whereas, for the SU(3) symmetry, 
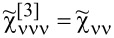
. The second important result is the transition from *F* = +1 to *F* = −1 for the empty and fully occupied states, where the total current is suppressed at the boundary (*I* = 0, bright white horizontal line). In the region where *F*_K_ dominates, we observe a characteristic vertical white line; along this line, the nonlinear current vanishes *I*_K_ = 0. This white line is surrounded by a zeroing contour of nonlinear noise *S*_K_ = 0 (black envelopes at the boundaries 1*e*/0*e* and (*N* − 1)*e*/*Ne*).

### Susceptibility and entropy

**Figure 6:**
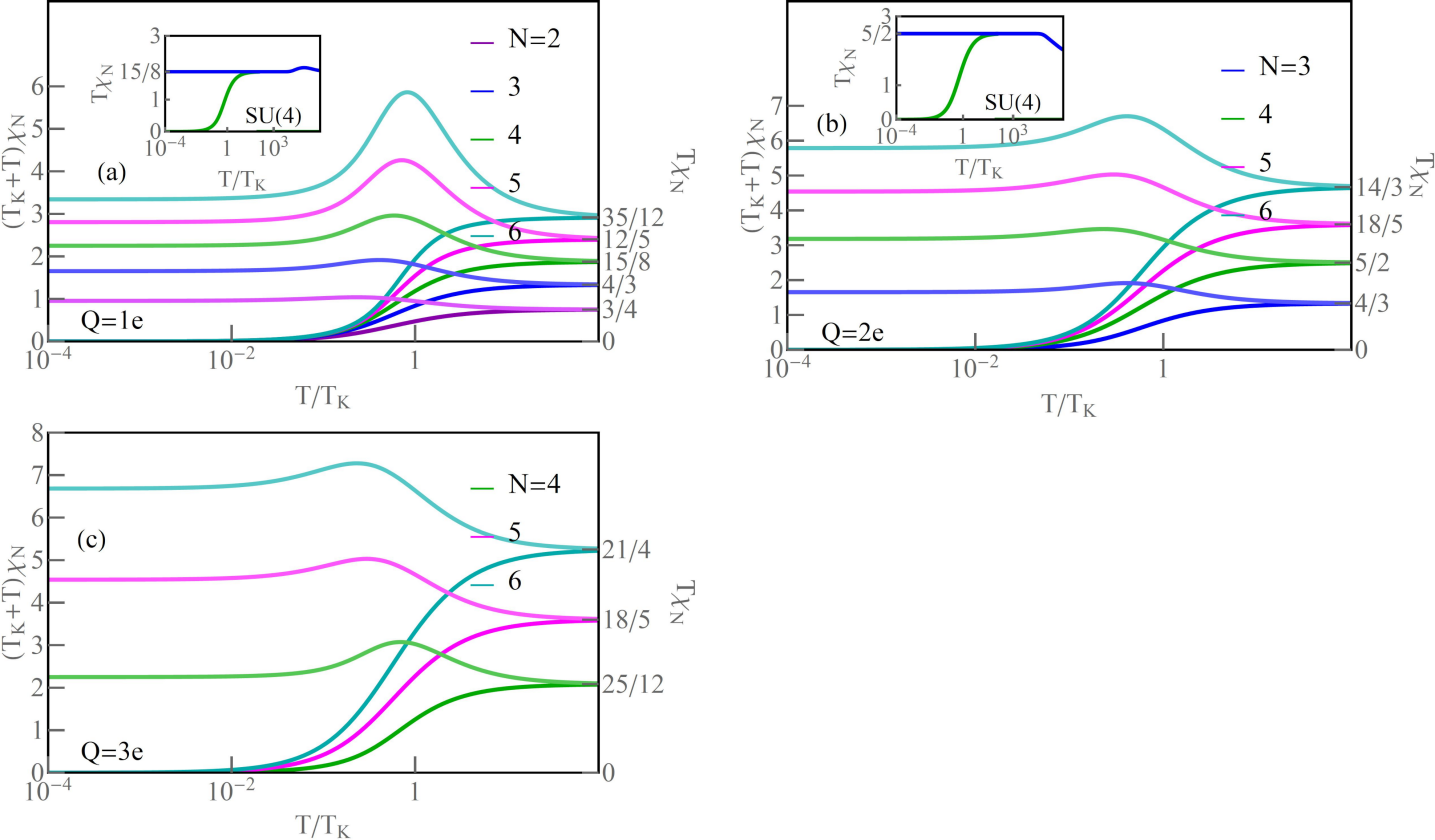
(a–c) Generalized spin *T*χ*_N_* and (*T*_K_ + *T*)χ*_N_* versus *T*/*T*_K_ (dark and light lines). The insets illustrate temperature dependencies of isolated and screened SU(4) spin for *Q* = 1*e* and *Q* = 2*e*, respectively (blue and green lines) (*U* = 3, Γ = 0.025, *V* = 0).

**Figure 7:**
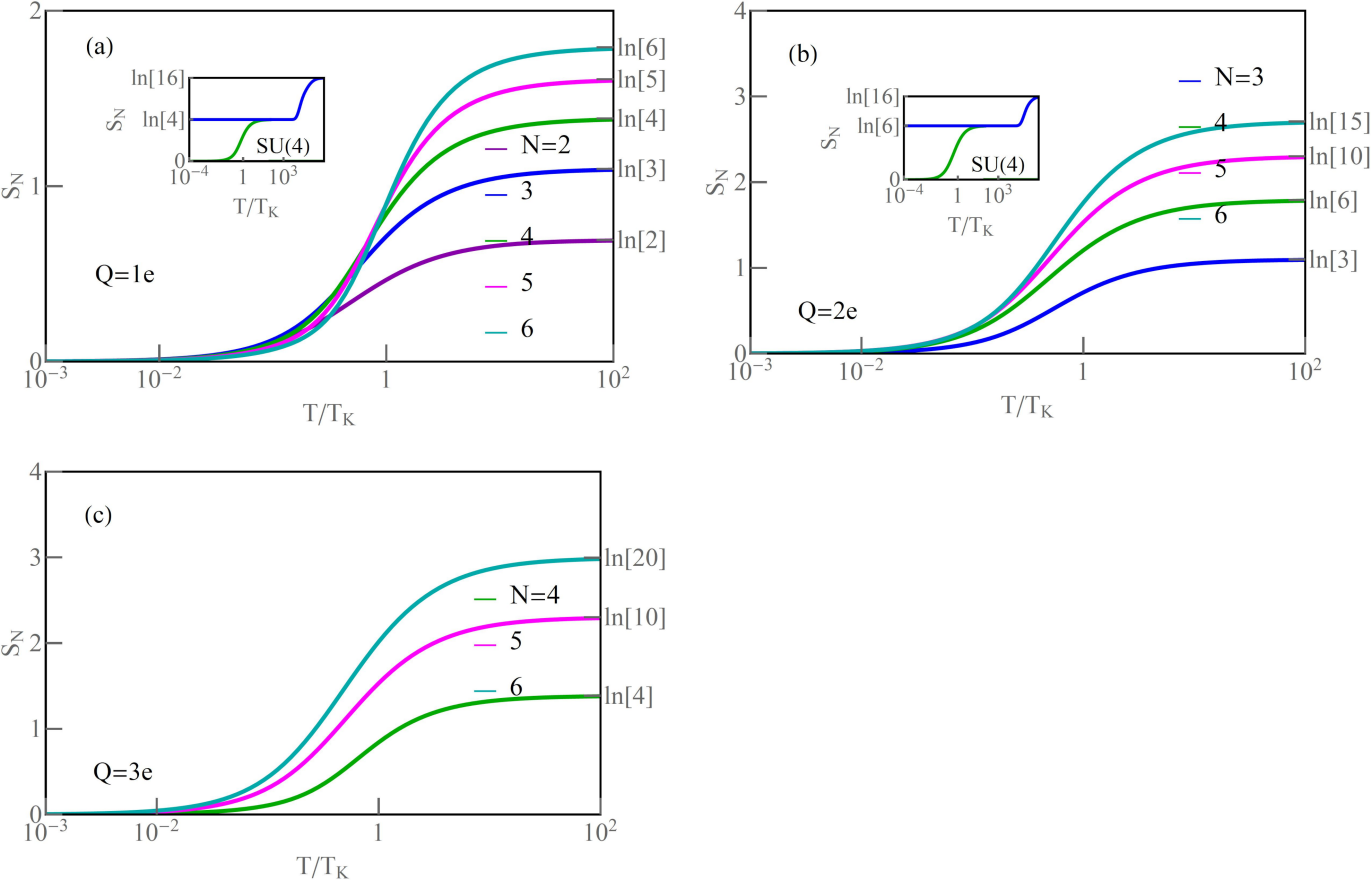
(a–c) The impurity entropies *S_N_* versus *T*/*T*_K_ for selected charge regions *Q* = 1*e*, 2*e*, 3*e*. The insets present temperature-dependent entropy *S_N_*_=4_ of isolated and screened SU(4) impurities (blue and green lines) (*U* = 3, Γ = 0.025, *V* = 0).

Generalized spin susceptibility and entropy provide direct, thermodynamic evidence for the formation of a Kondo singlet. Figure 6 and Figure 7 present the temperature dependencies of susceptibilities, generalized moments, and entropies for different SU(*N*) systems and various occupancies. *T*χ*_N_* (dark curves in Figure 6), where 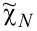 denotes the generalized spin susceptibility, illustrates the fluctuation with increase of the temperature of the squared generalized SU(*N*) spins defined by Equation 13. Local Coulomb repulsion favors single occupancies of spin-orbitals, and local SU(*N*) moments form at high temperatures. This fact is illustrated by the saturation of the continuous lines *T*χ*_N_* in Figure 6a–c to the values of squared generalized local SU(*N*) spins *T*χ*_N_* = (*N* + 1)*NQ*_ν_(1 − *Q*_ν_)/2. The saturation values are described by Equation 21. For SU(2) and *n* = 1, the corresponding value is 3/4, for SU(3) 4/3, for SU(4) 15/8, for SU(5) 12/5, and 35/12 for SU(6). Similarly, for *n* = 2 and *n* = 3, the saturation values can be read from Figure 6b,c.

The light lines in Figure 6 show temperature dependencies of SU(*N*) susceptibilities. As it is seen, they evolve from Curie-type behavior for high temperatures to Pauli-like behavior, temperature-independent at low temperatures [107]. For example, we show in the insets of Figure 6a,b the exemplary comparison in a wide temperature range of the moments for an isolated SU(4) system (blue lines) with the moments of an SU(4) QD coupled to electrodes (green lines). Around *T* ∼ 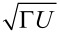, there is a transition from the range of local moment on the dot to the free-orbital regime, where probability of occupation of all spin-orbitals is equal [108–110]. At low temperatures, below *T*_K_, the role of Kondo fluctuations becomes dominant, and, for *T* → 0, the moment of the dot is dynamically screened by electrode electrons and a many-body SU(*N*) Kondo singlet is formed. Apart from observations in transport, quite recently also the progressive screening of the spin of a single Anderson impurity (SU(2)) was directly measured [107].

Examples of the temperature dependence of the moments for SU(4) symmetry are illustrated by solid (green) lines in Figure 6a,b. They tend to zero for *T* → 0. For high temperatures, moments saturate to a value *T*χ*_N=_*_4_ = 15/8. In this specific case, spin σ, orbital moment τ, and multipole στ are also quenched. In general, however, the screening of the SU(*N*) moment is not necessarily accompanied by quenching of conventional spin or orbital moments. This is the case, for example, for the discussed SU(3) or SU(5) symmetries. Thermodynamic behavior of SU(*N*) moments coupled to electrode electrons is also reflected in the temperature dependencies of entropy *S_N_*. The Kondo ground state is a singlet; therefore, as it is seen in Figure 7, for *T* → 0, *S_N_* → 0. The rise of temperature causes an increase of entropy. For *T > T*_K_, the local moment becomes unscreened and free; for *n* = 1, the entropy is determined by *N* lowest, degenerate single electron states, and *S_N_* = *k*_B_ln[*N*]. For *n* = 2, the low-temperature entropy is determined by the lowest degenerate two-particle states and, for *n* = 3, by the degenerate three-particle states. Entropy expressed by the slave boson amplitudes is 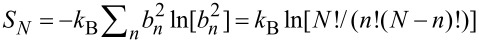. For even higher temperatures, more states become excited, allowing thermal energy to be more widely spread. For *T* ≫ 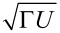, all *N*^2^ states of the SU(*N*) system are thermally accessible, and entropy reaches the value 2*k*_B_ln[*N*]. Examples of entropies in a wide temperature range are presented in the insets of Figure 7a,b.

## Conclusion

We have investigated transport and thermodynamic properties of a multilevel degenerate QD symmetrically coupled to leads. In the analysis of correlations, we innovatively combine the description of the equilibrium Kondo state based on generalized slave boson techniques (SBMFA) with a microsopic Fermi liquid approach for high voltages, the parameters of which were derived from slave boson fields. Our study is the first systematic discussion of all SU(*N*) Kondo states to high orders up to *N* = 6. We put special attention on the analysis of the shot noise. Compared to the conductance, shot noise is more difficult to investigate experimentally, but its examination is essential because it provides extended information about dynamics of electron transfer and correlations. The systems under investigations are characterized by the SU(*N*) group. For *N >* 2, spin is replaced by generalized spin, which refers to the representation of the SU(*N*) Lie group. The square of the SU(*N*) spin, *S*^2^, and its longitudinal component *S*_Z_ commute with the Hamiltonian; hence, the invariant subspaces can by classified by the quantum numbers *S* and *S*_Z_. For even *N*, many-body resonances appearing in the particle–hole symmetric point of SU(*N*) structures represent Kondo states, while, for odd *N*, these are charge resonances. For half-filling, both types of resonances are centered at the Fermi level; therefore, perfect transmission occurs, the partial conductance *G* reaches the unitary limit at low temperatures (*G* = *e*^2^/*h*), and the total conductance is *G* = *Ne*^2^/*h*. The linear Fano factor, also directly related to transmission, vanishes at *E*_F_ in this case. For this occupancy, the Kondo temperature reaches its lowest value. For even *N*, Kondo resonances hardly move with the change of gate voltage, and, hence, plateaus are observed in the dependencies of conduction and Fano factors on the gate voltage. For odd *N*, charge resonances broaden and shift already for small changes of gate voltage, and peaks of conductance or dips of linear Fano factor are seen. The Kondo resonances away from half-filling are shifted relative to the Fermi level; hence, the transmissions decrease and, consequently, the values of conduction plateaus decrease and, correspondingly, *F*_0_ plateaus rise. Also, a decrease of *T*_K_ is observed for *n* ≠ *N*/2. Important information is the strength of correlations and, therefore, we also discussed Wilson ratio *W*_νν'_, which quantifies it. If this ratio is different from 1, it indicates that the correlations are significant. In Kondo systems, where localized electrons interact strongly with conduction electrons, the Wilson ratio can be enhanced compared to the Fermi gas value. This enhancement is associated with strong fluctuations of the generalized spin and the formation of a Kondo resonance. In the Kondo regime, charge susceptibility in the limit *U* → ∞ is zero or almost zero for strong interactions; thus, Wilson ratios take the universal values characteristic for a given *N* and weakly depend on the occupation sector. Interestingly, *W*_νν'_ decrease in charge fluctuation regions, but they still retain significant values, indicating the importance of correlations also in these areas. The strength of correlations in a system tends to decrease as the degree of degeneracy increases because more degenerate states allow for compensatory changes to disturbances introduced by interactions. In the limit *N* → ∞, the quasiparticles become independent, *W*_νν'_ → 1. Approaching empty or fully occupied regions, the Wilson ratios also tend to unity for any *N*.

Screening of magnetic moment by conduction electrons is a key process of the spin-Kondo effect. In this paper we have considered a more general case, the effects of strong coupling of *N*-dimensional generalized spin with conduction electrons. To illustrate quenching of the generalized moments, we plotted generalized susceptibilities and entropies for each of the examined symmetries. As the temperature is lowered, the susceptibility becomes Pauli-like and temperature-independent, and the effective moment tends to zero. In the Kondo states, the entropy associated with the localized moment is reduced due to the formation of a Kondo singlet resulting from screening of the SU(*N*) spin. Above *T > T*_K_, the entropy reaches *S_N_* = *k*_B_ln[*N*!/(*n*!(*N* − *n*)!)], and for *T* = 0 it vanishes. Shot noise measures out-of-equilibrium current fluctuations. As we mentioned earlier in the text, the linear Fano factor corresponds to partition noise, that is, it reflects fluctuations related to the partition of the scattered particles. Nonlinear shot noise arises from the fluctuations in the number of discrete charge carriers passing through a system. It results from interactions between quasiparticles. The important thing is that nonlinear shot noise contains signatures of 2*e* scattering, which are not seen in the conductance. Our study confirms a conviction of previous authors [82] that taking into account the nonlinear contribution can completely change the picture of fluctuations, in particular whether they are super-Poissonian in nature or sub-Poissonian. These characteristics have been studied by us based on the Fermi liquid theory, assuming dressed, non-interacting pseudofermions defined within SBMFA as quasiparticles. Fermi liquid parameters describing quasiparticle interaction were expressed by two- and three-body correlation functions. Competition of these correlations going beyond linear contribution to the shot noise can determine the nature of nonlinear noise. The dominance of particular scattering processes changes as the energy or chemical potential moves away from the symmetry point. We illustrated on the example of the SU(4) group the general properties of correlations; the two-body correlations are symmetric, whereas three-body correlations are antisymmetric with respect to electron–hole symmetry point. This behavior is a consequence of the fermion nature of particles and the related antisymmetry of the wave functions. Consequently, the three-body residual interactions have a different impact on the behavior of electrons and holes. The three-body correlations vanish at the e–h symmetric point and are very weak in its neighborhood; their absolute value is significant in the Kondo regions beyond half-filling. In contrast, the two-body correlations are positive and dominate for *n* = *N*/2; they get weaker away from half-filling so that three-particle residual interactions dominate over two-particle interactions for regions with distinct differences in number of electrons and holes. Our calculations point out that the nonlinear Fano factor tends to decrease with increasing distance from the e–h symmetry point. The current fluctuations of carriers with dominant population become more correlated with the total current, while those of the remaining carriers become less correlated. This leads to less noisy current flow. The rank of the unitary group has also a significant impact on nonlinear shot noise. Higher-symmetry Kondo states tend to have increased shot noise compared to lower-symmetry states due to enhanced many-body correlations involving more components of generalized spin.

The conclusions drawn in this paper can be easily adopted for the case of other structures described by the same Hamiltonian, for example, degenerate impurities, materials with multiple valleys, or cold-atom arrangements. Apart from enriching the knowledge about correlations, the discussed problems are of importance for quantum computing and other quantum technologies. The multidimensional generalized spin (qudit) offers greater information capacity and algorithmic efficiency compared to traditional spins. The Kondo effect provides a powerful, tunable platform to observe and control entanglement. Operating qudit involves carefully tuning the system (e.g., by magnetic or electric fields) to be near or far from the Kondo regime, depending on the desired operation (e.g., initialization, entanglement, or readout). The main limitation of the possible applications of the ordinary spin-SU(2) Kondo effect in nanoobjects is its ultralow Kondo temperature. The increase of SU(*N*) symmetry removes this problem because *T*_K_ is growing exponentially with *N*, and higher temperatures open up possibilities for the practical use of these systems. Noise is a key area of interest. By studying changes in the shot noise, researchers can infer the level of decoherence occurring within the quantum system. This knowledge is extremely important because decoherence is a critical factor that affects the stability and reliability of quantum information.

## Appendix A. Slave Boson Formulation

For the discussion of correlation effects, we use the generalized finite-*U* slave boson mean-field approximation (SBMFA) of Kotliar and Ruckenstein (K-R) [87]. We present here only the basics of the slave boson formalism using a system with *N* = 6 levels as an example. For lower multiciplicities (*N <* 6), the formalism is analogous, but simplified due to the smaller number of describes states, which allows for the use of fewer bosons. This reduces the number of self-consistent minimization equations. For SU(6), we introduce a set of boson operators for each electronic configuration of the system. The auxiliary bosons {*e*, *p*, *d*, *t*, *f*, *q*, *s*} project onto empty, single, double, triple, fourfold, fivefold, and fully occupied states. The single occupation projectors *p_i_*_σ_ (*q_i_*_σ_) are labeled by orbital or site and spin numbers. Among double (*d_i_*,*d_ij_*_σσ'_) and fourfold (*f_i_*,*f_ij_*_σ_) occupancy bosons, two classes can be distinguished. The first corresponds to the occupancy of a single orbital by two electrons (*d_i_*) or two holes (*f_i_*), and the second describes the double occupancy of electrons (*d_ij_*_σσ'_) or holes (*f_ij_*_σσ'_) in different orbitals. The three-electron occupations are represented by twelve bosons (*t_ij_*_σ_) corresponding to double occupation of one of the orbitals and single occupation of another, and eight (*t*_σσ'σ''_) project onto states with single occupation on the orbital. For SU(6) symmetry, the use of seven independent slave bosonic (SB) operators is sufficient; in general, for SU(*N*), their number is (*N* + 1). Apart from slave bosonic operators, one also introduces in the SB approach auxiliary fermionic operators, in terms of which the physical electron operators *d_i_*_σ_ are expressed by *z_i_*_σ_*f_i_*_σ_, where *z_i_*_σ_ is the transfer bosonic like-operator, which modifies coupling to the leads:


[37]

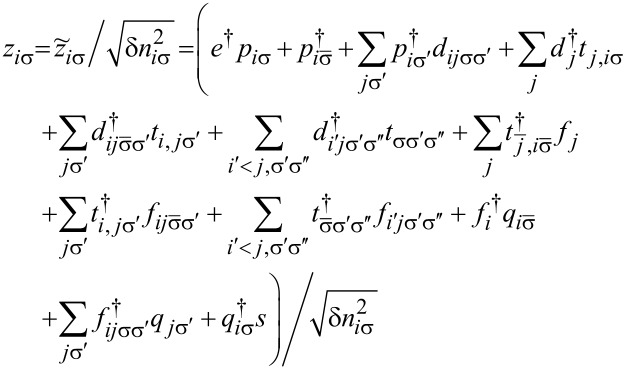



*f_i_*_σ_ represents the pseudo-fermionic operators that renormalize orbital–lead hybridization, 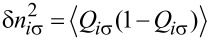 is the square of the orbital-spin fluctuations number, and


[38]

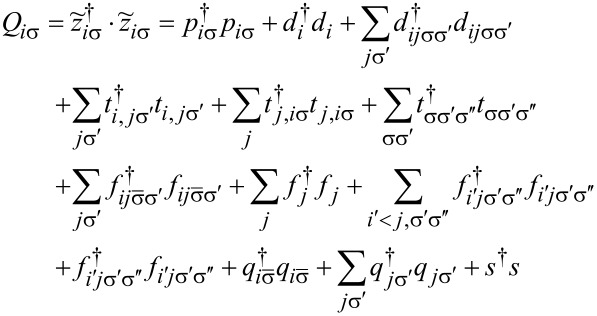



denotes the charge operator. The tunneling (transfer) operator 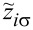 is normalized by the variance of orbital-spin occupancy number 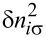. The above form of effective resonant line narrowing factors *z_i_*_σ_ are chosen in order to obtain the correct MFA limit in the uncorrelated case. To eliminate additional unphysical states introduced by SB representation, one supplements the SB Hamiltonian with conditions of charge conservation *Q_i_*_σ_ and completeness relation expressed by equating the sum of bosonic amplitudes with the unit operator


[39] $$\begin{array}{c} I\;=\;e^{†}e\;+\text{​}\sum_{i\sigma }p_{i\sigma }^{†}p_{i\sigma }\;+\text{​}\sum_{i}d_{i}^{†}d_{i}\;+\text{​}\sum_{ij\sigma \sigma^{\prime }\text{​},i<\text{​}j}d_{ij\sigma \sigma^{\prime }}^{†}d_{ij\sigma \sigma^{\prime }}\\ +\text{​}\sum_{ij\sigma ,i\neq j}t_{i,j\sigma }^{†}t_{i,j\sigma }\;+\text{​}\sum_{\sigma \sigma^{\prime }\sigma^{\prime \prime }}t_{\sigma \sigma^{\prime }\sigma^{\prime \prime }}^{†}t_{\sigma \sigma^{\prime }\sigma^{\prime \prime }}\;+\text{​}\sum_{i}f_{i}^{†}f_{i}\\ +\text{​}\sum_{ij\sigma \sigma^{\prime },i<j}f_{ij\sigma \sigma^{\prime }}^{†}f_{ij\sigma \sigma^{\prime }}\;+\text{​}\sum_{i\sigma }q_{i\sigma }^{†}q_{i\sigma }+s^{†}s. \end{array}$$

These constraints are built into the SB Hamiltonian by introducing Lagrange multipliers λ and λ*_i_*_σ_. The corresponding extended K-R Hamiltonian then reads:


[40]

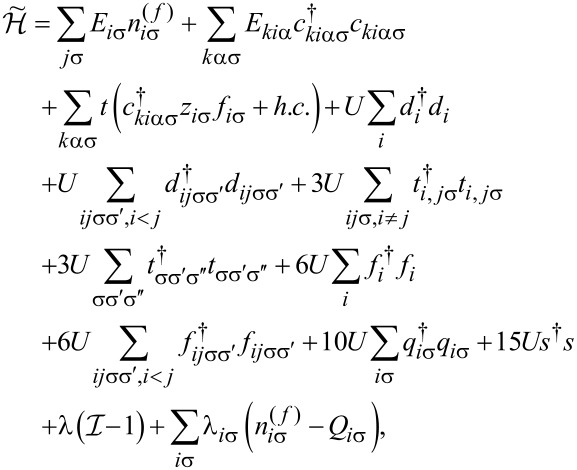



where 
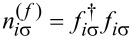
 is the pseudofermion occupation number operator [87]. The MFA ground state is found using the saddle-point approximation (K-R), in which all boson fields are replaced by their expectation values, found from the condition that 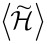 has an absolute minimum as a function of variables {*b_n=_*_1..64_} = {*e*, *p_i_*_σ_, *d_i_*, *d_ij_*_σσ'_, *t_i,j_*_σ_, *t*_σσ'σ''_, *f_i_*, *f_ij_*_σσ'_, *q_i_*_σ_, *s*} and the Lagrange multipliers λ, λ*_i_*_σ_. In this way, the problem is formally reduced to the effective quasiparticle model with renormalized hopping integrals and renormalized dot energies. The resulting self-consistent minimization equations read:


[41]

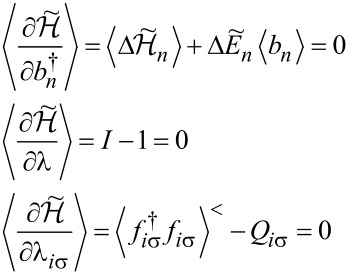



where


[42]

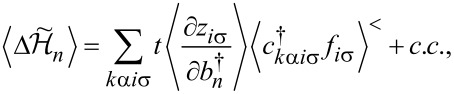




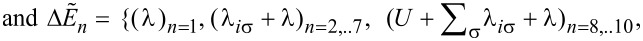

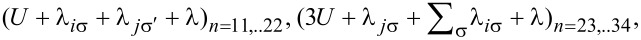

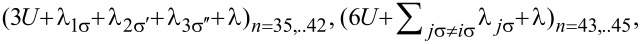

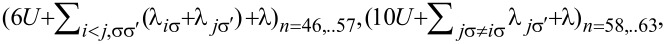

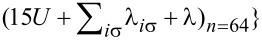
 correspond to effective corrections of the dot energies for different occupations. The correlators can be expressed by the corresponding non-equilibrium Green’s functions:


[43]

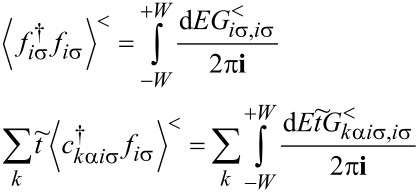




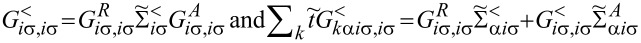
 are the non-equilibrium Green’s functions that can be found by the equations-of-motion method applied to the SB Hamiltonian (Equation 2). 
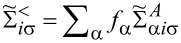
 and 
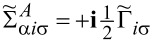
 are, respectively, lesser and advanced self-energies. The retarded and advanced Green’s functions in channel ν are 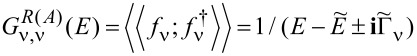, where the poles determine position 
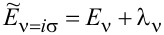
 and width of the quasiparticle resonance 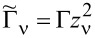. The corresponding characteristic resonance temperature is 
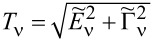
. The renormalized level position and the width also specify the located charge and, thus, also the phase shift (δ_ν_) at a given orbital *Q*_ν_ = δ_ν_/π. The link between the complex pole of the Green’s function, charge, and characteristic temperature can be expressed as follows [89]:


[44]

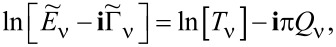



or equivalently:


[22]

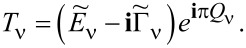



In the mean-field approximation, the free energy corresponding to the Hamiltonian in Equation 2 is a sum of slave-boson free energy 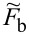 and fermionic contribution 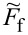 (
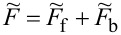
). Using the Matsubara Green’s function the fermionic and bosonic free energies can be written as:


[45]

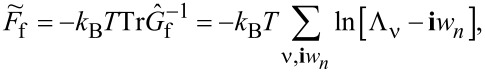



where 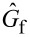 is the full f-electron propagator in the presence of the Bose field (represented by the Green’s function matrix in general), *iw_n_* are the Matsubara frequencies, and 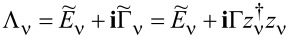 are the complex poles of the quasiparticle Kondo resonance. After integration of Equation 16 on the complex plane, one gets [111]:


[46]

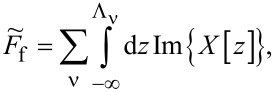



where 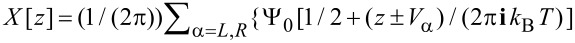 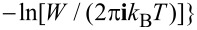 and Ψ_0_ is the digamma function [89]. The slave boson contribution is:


[47]

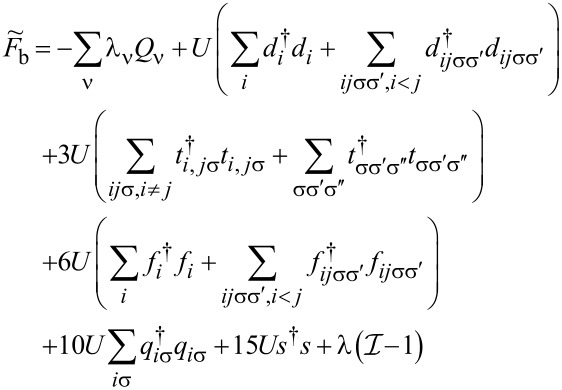



The phase shift δ_ν_, which, according to Friedel sum rules, determines the elastic scattering, is linked with the corresponding partial density of states at the Fermi level as follows 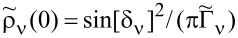. At zero temperature, δ_ν_ is also related to the occupation number by:


[7]

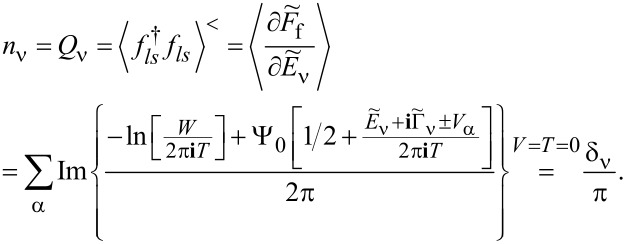



Similarly, static two-body susceptibilities 
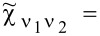
 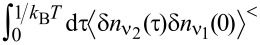 and three-body correlation functions 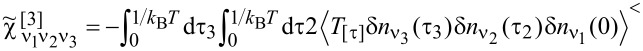 are expressed through derivatives of the free energy with respect to site energies. δ*n*_ν_ denote deviations from the ground state distribution δ*n*_ν_ ≡ *n*_ν_ − ⟨*n*_ν_(0)⟩.

The diagonal fermionic susceptibilities at low temperatures are given by [89]:


[8]

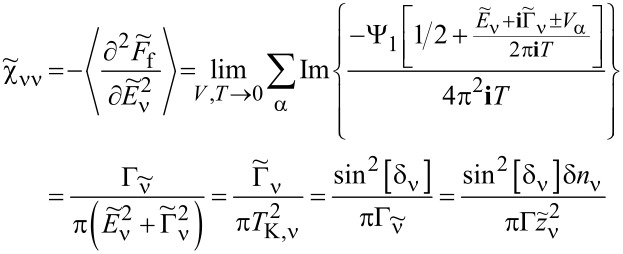




[48]

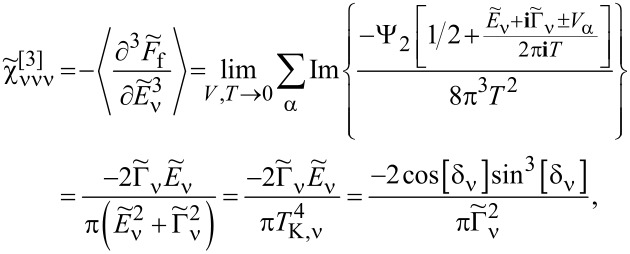



where Ψ_1_*_(_*_2_*_)_*(*z*) are the first and second derivative of the digamma function Ψ_0_. Equation 8 and Equation 48 determine correlators within the SBMFA approach. Although quasiparticles in this approximation are formally treated as non-interacting, some of the effects of interaction are encoded in this approach by renormalization of parameters.

**Figure 8:**
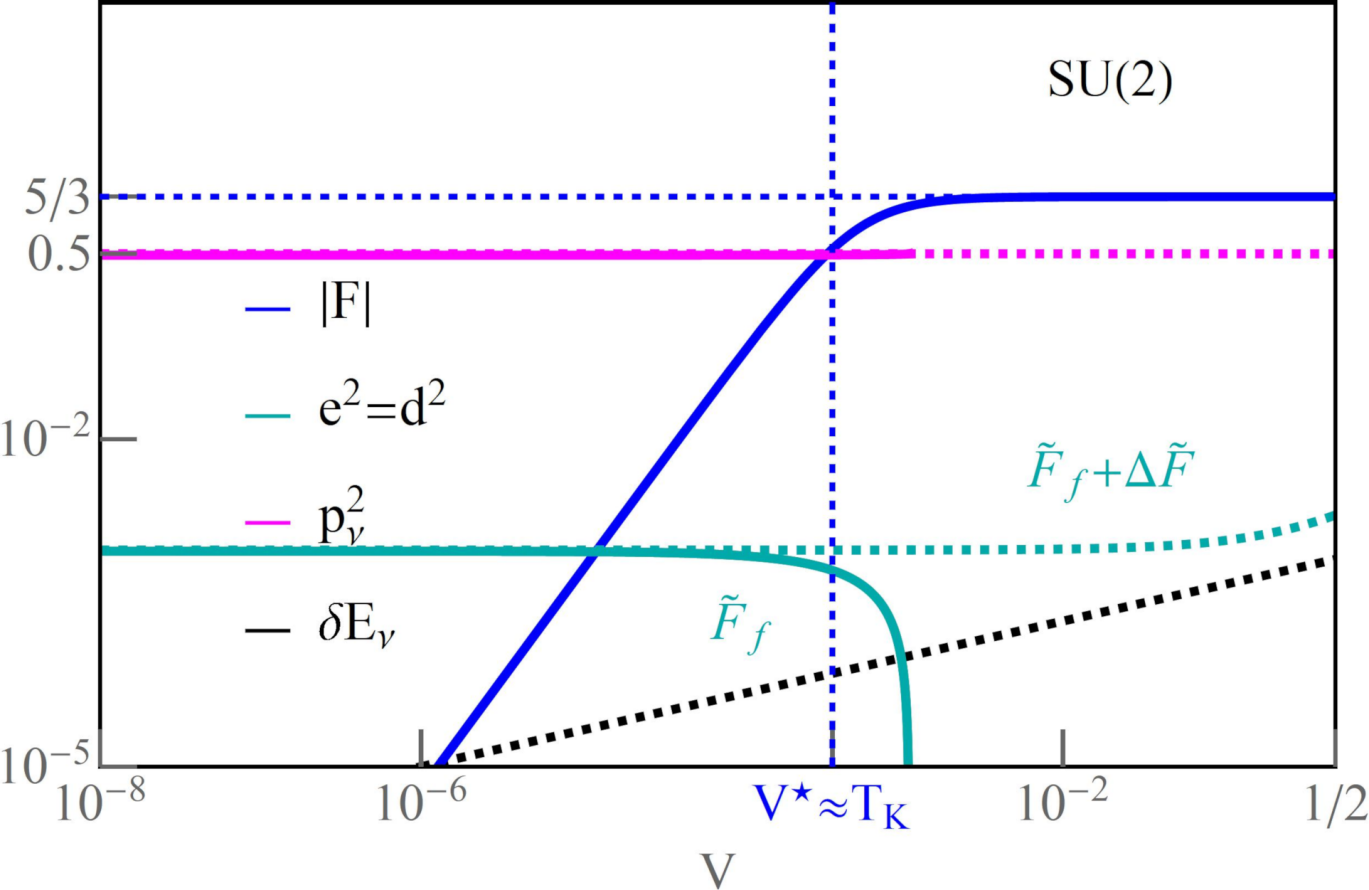
Slave boson amplitudes 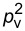, *d*^2^ and the fluctuation parameter δ*E*_ν_ versus bias voltage (magenta dark cyan and black lines). Solid and dashed curves represent, respectively, the self-consistent calculations without and with FL correction (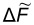) to free fermion energy 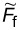. *T*_K_ is shown on the *V*-axis. Blue line illustrates total |*F*| versus V for SU(2) Kondo state (*U* = 3, *T* = 0, *E*_d_ = −*U*/2, Γ = 0.05).

For discussion of the out-of-equilibrium case (*V* ≠ 0), we extended the MFA approach to slave bosons by including into the self-consistent minimization equations the free energy supplemented by the Fermi liquid interactions term 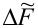 (Equation 23). We integrated 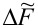 putting 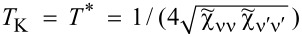 and taking the fluctuation of the occupation number δ*n*_ν_ = 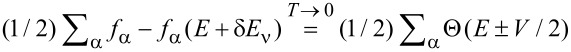 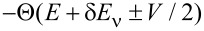 (*f*_α_ is the Fermi–Dirac distribution and Θ is the Heaviside function). In this generalized procedure appeared the new additional minimization parameter δ*E*_ν_ (fluctuation of energy levels of FL quasiparticles). The corresponding minimization equation is 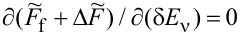, and it supplements Equation 41 and Equation 42. As is seen in Figure 8, the SBMFA solutions do not exists for *V* ≫ *T*_K_. The proposed method interpolates slave boson approach for high voltages. This approach extends the existence of auxiliary bosons in the voltage range *U* ≫ *V > T*_K_ (Figure 8). This method is an alternative proposition to the problem of fluctuations in FL theory different from the earlier schemes presented, for example, in [90,111–112]. The condition ∂^2^|*F*|/∂*V*^2^ = 0 determines the characteristic voltage 
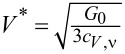
, for which the picture of non-interacting Kondo particles breaks down and Landauer’s description no longer applies.

## Appendix B. The Analytical Expression for the Kondo Temperature in the SBMFA Formalism

Here we only sketch the main points of the derivation, a more detailed analysis, but only for the special case of SU(4) symmetry can be found in Appendix A of [113]. We restrict ourselves to the fully symmetric SU(*N*) Kondo state. In this case, the number of independent boson operators reduces from 2*^N^* to *N* + 1. To derive an approximate analytical formula for *T*_K_ applicable in a given charge sector {*Q*} and for simplicity, we take into account the relationship of the minimization Equation 3 for a given charge sector {*Q*} with analogous equations, but only from the nearest sectors {*Q* ± 1}. This simplification means that the dynamics within a given charge sector *Q* = *ne* is influenced by fluctuations (*n* − 1)*e* ↔ *ne* and *ne* ↔ (*n* + 1)*e* alone. Let us denote the difference of two self-consistent equations for *n* and *n* − 1 and for given *N* by 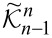
(*N*):


[28]

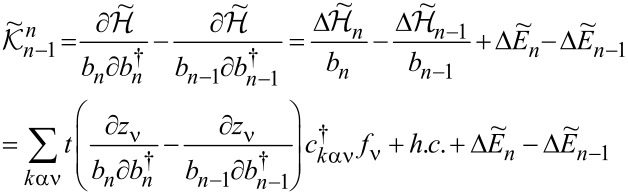



For *V* = 0 and *T* = 0 the above formula is simplified to:


[49]

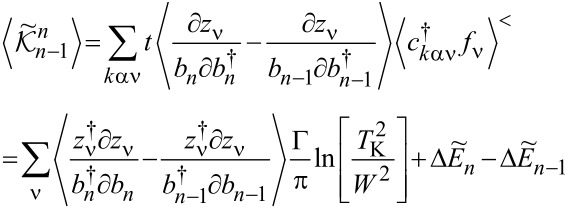



In the unitary limit, λ_ν_ ≈ *E*_d_ and then:


[29]

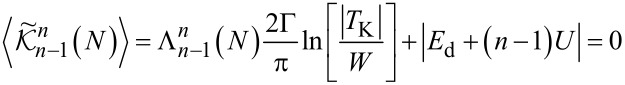



and similarly


[50]

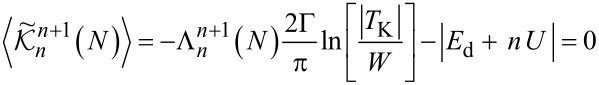



where the coefficients 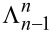
(*N*) and 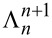
(*N*) are assigned to the virtual transitions between neighboring charge states. For *N* = 2 
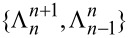
 = {4,4}, *N* = 3 
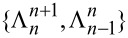
 = {6,9/2}, {9/2,6}, and for *N* = 4 {8,16/3}, {6,6}, {16/3,8}. For SU(5) and SU(6) Kondo symmetries, 
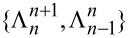
 = {10,25/4}, {15/2,20/3} and {12,36/5}, {9,15/2}, {8,8}, respectively. For infinite *U*, 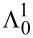 is different from zero, and 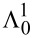 = *N*^2^/(*N* − 1); in particular, for the SU(4) symmetry, 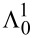 = 16/3 [113]. Comparing the left sides of Equation 40 and Equation 41 allows us to determine the SU(*N*) Kondo temperature in selected charge sectors *Q* = *ne*.


[51]

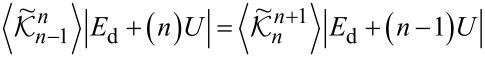



From Equation 42 it follows:


[52]

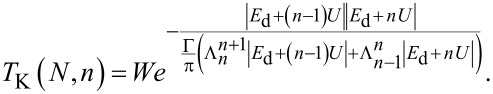



The Kondo temperature is expressed in terms of the bare parameters of the Anderson model. Equation 43 generalizes the K-R SBMFA expression for SU(2) onto the systems of higher symmetries SU(*N*), *N >* 2. For infinite *U*, Equation 43 is simplified to:


[34] $$T_{\text{K}}\left(N\right)=We^{-\frac{\left|E_{\text{d}}\right|}{\frac{\Gamma }{\pi }\Lambda_{0}^{1}\left(N\right)}}.$$

For *N* = 2, the above formula differs from Coleman’s SBMFA expression [114–115] and from the commonly cited formula for *T*_K_ derived from Schrieffer–Wolff transformation [24] by a factor of two in the denominator of the exponent. The difference is related to disparate representation of charge fluctuations in the K-R and Coleman approaches (different number of SB operators). For *N* = 4, Equation 44 has been derived earlier by us [113]. The Kondo temperature increases with an increase of degeneracy. This is most clearly seen in the limit of infinite *U*, where the Kondo temperature scales by only one coefficient in the standard exponential dependence, namely, 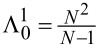 [15,113]. For high degeneracy, 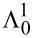 → *N*. The increase in *T*_K_ along with the increase in degeneration opens a window to Kondo system applications. The increase of *T*_K_ with *N* is observed in differential conductance spectroscopy [11,29,101], where the width of the quasiparticle resonance increases with higher *N* [15].

We can express two- and three-body correlation functions using the Kondo temperature as follows:


[35]

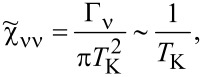




[30]

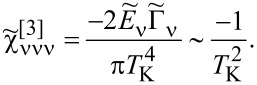



Based on the above equations, we find 
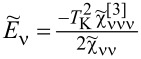
, and we can write equivalently the Kondo temperature (Equation 43) as a function of not only two-body, but also three-particle, correlators:


[53]

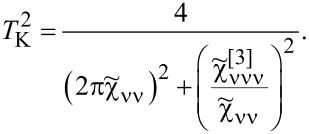



## Appendix C. Susceptibilities and Wilson Ratio of an isolated N-QD

The slave boson Hamiltonian of the N-QD in the atomic limit (*t* = 0) reads:


[54]

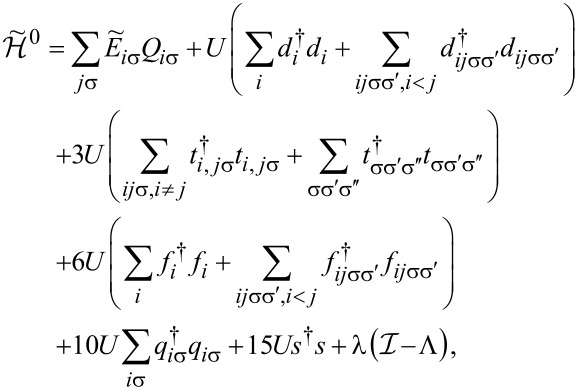



The corresponding partition function is:


[55]

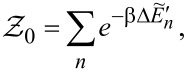



where β = 1/*k*_B_*T*, 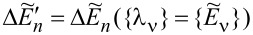 and 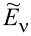 is defined in the main text. The probability distribution can be determined by the assignment:


[56]

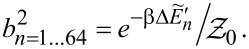



The free energy of an isolated N-QD is *F_(_*_0_*_)_* = −(1/β)ln(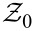). Now, we present formulas for susceptibilities. For clarity and brevity of expressions, we illustrate the derivation of formulas only for the simplest case of SU(2) symmetry, which requires the use of four slave bosons. For higher symmetries, the considerations are completely analogous. For SU(2) symmetry, the set of SB operators is 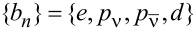 and 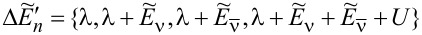. Susceptibilities are given by:


[57]

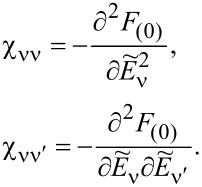



Using the inverted relation to Equation 29 we can write:


[58]

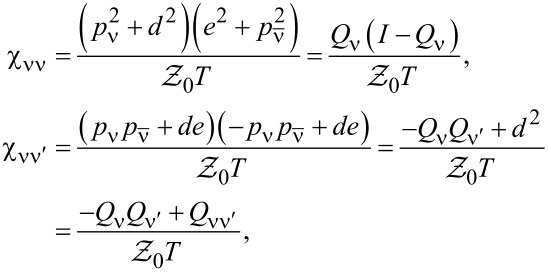



where 
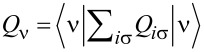
 is the charge in state ν and *Q*_νν'_ = 
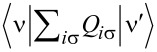
 is the inter-state charge correlator.

The Wilson ratio


[59] $$W_{\nu \nu^{\prime }}^{\left(0\right)}-1\equiv -\frac{\chi_{\nu \nu^{\prime }}}{\sqrt{\chi_{\nu \nu }\chi_{\nu^{\prime }\nu^{\prime }}}}$$

can be expressed using Equation 51 as follows:


[31] $$W_{\nu \nu^{\prime }}^{\left(0\right)}-1=\frac{Q_{\nu }Q_{\nu^{\prime }}-Q_{\nu \nu^{\prime }}}{\sqrt{Q_{\nu }\left(I-Q_{\nu }\right)Q_{\nu^{\prime }}\left(I-Q_{\nu^{\prime }}\right)}}$$

It is important to note that, while the corresponding bosonic expressions for the susceptibilities differ for individual symmetries because the sets of auxiliary bosons are different, the formulas for susceptibilities given by the charges and charge correlators have a general form, applying to any *N*. According to the argument presented in the main text, we adopt the above formula for *W*_νν'_ (Equation 51) also for the case when electrodes are weakly linked to the dot (see Equation 14).

**Figure 9:**
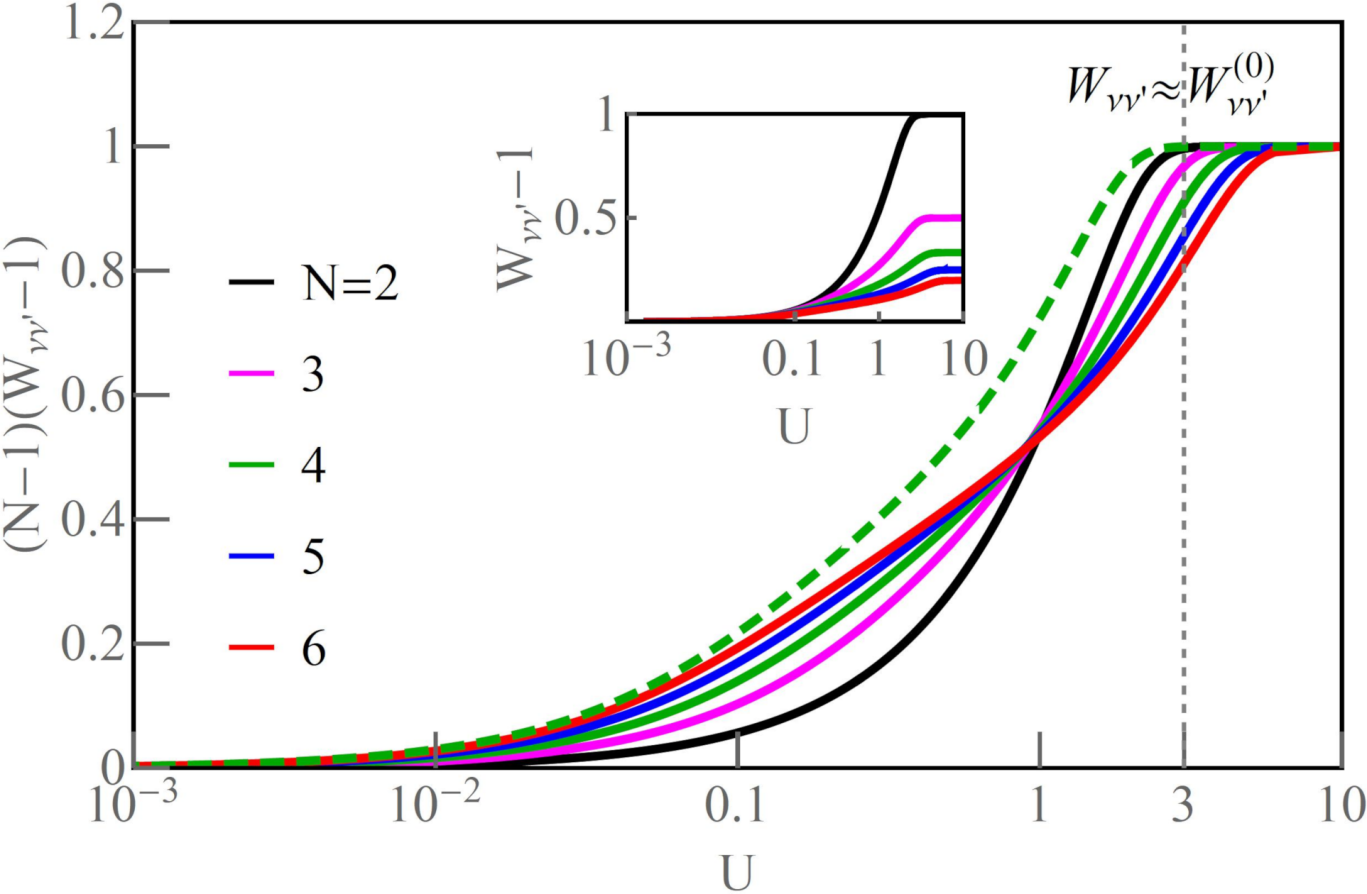
Generalized Wilson ratio (*N* − 1)(*W*_νν'_ − 1) as a function of Coulomb interaction *U* in the 1*e* charge region. The inset shows *W*_νν'_ − 1 versus *U* (Γ = 0.05). The green dashed line shows the generalized Wilson ratio of the SU(4) Kondo state for Γ = 0.025. The dashed vertical gray line represents *U* used in calculations.

Figure 9 presents the dependence of the generalized Wilson ratio on the Coulomb interaction parameter *U* exemplarily for *Q* = 1*e*. For high values of *U*, *W*_νν'_ − 1 saturates and takes the values 1/(*N* − 1) (inset in Figure 9), which agrees with the results of NRG and perturbation theory calculus [80,84,96]. As we have shown earlier, in the regime of strong coupling, the Wilson coefficient depends only on symmetry (*N*); due to the smallness of the quasiparticle amplitude 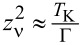, it is well defined by the charges *Q*_ν_ and the inter-charge correlator *Q*_νν'_ (Equation 31). Lowering the coupling value with the electrodes Γ effectively shifts the saturation point (*N* − 1)(*W*_νν'_ − 1) toward lower *U* values.

## Appendix D. Nonlinear Shot Noise of FL Quasiparticles in SBMFA

As shown in [82–83], the nonlinear coefficients *c_V,_*_ν_ and *c_S,_*_ν_ of current and shot noise can be expressed by the following high-order correlations and phase shifts as follows:


[32]

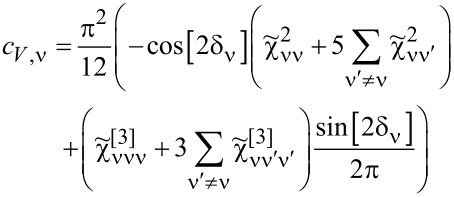




[33]

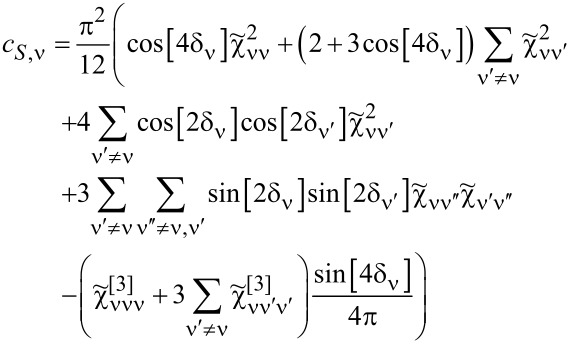



The nonlinear Fano factor *F*_K_ reads [82]:


[36] $$F_{\text{K}}=\frac{S-S_{0}}{2\left|e\right|\left(I-I_{0}\right)}=\frac{S_{\text{K}}}{2\left|e\right|I_{\text{K}}}=\frac{\sum_{\nu }c_{S,\nu }}{\sum_{\nu }c_{V,\nu }}.$$

Expressing two- and three-particle functions in Equation 32 and Equation 33 by slave-boson mean-field correlators, we can rewrite the total shot noise formula for SU(*N*) Kondo quasiparticles up to *V*^3^ as follows:


[60]

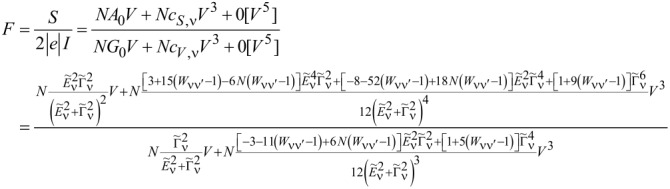



Equation 60 explicitly shows the dependence of |*F*| on Kondo resonance characteristics (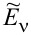 and 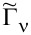), degeneracy (*N*), and Wilson ratio *W*_νν'_. When *W*_νν'_ = 1, the interaction corrections disappear, and the system behaves like a noninteracting system of Kondo particles.

## Data Availability

All data that supports the findings of this study is available in the published article and/or the supporting information of this article.
